# Supplementary material for: Excited-State Polarizabilities: A Combined Density Functional Theory and Information-Theoretic Approach Study
Source: Molecules. 2023 Mar 12;28(6):2576. doi: 10.3390/molecules28062576 (PMC10058485; doi:10.3390/molecules28062576)
Supplement: Supplementary file 1 [file molecules-28-02576-s001.zip › molecules-2223866-supplementary.pdf]

Supplementary information for

Excited-State Polarizabilities: A Combined Density Functional Theory and Information-Theoretic Approach Study

Dongbo Zhao[1], Xin He[2], Paul W. Ayers,[3,*] and Shubin Liu[4,5,*]

[1] Institute of Biomedical Research, Yunnan University, Kunming 650500, China

[2] Qingdao Institute for Theoretical and Computational Sciences, Shandong University, Qingdao 266237, China

[3] Department of Chemistry and Chemical Biology, McMaster University, Hamilton, ONL8S 4M1, Canada

[4] Research Computing Center, University of North Carolina, Chapel Hill, NC 27599-3420, USA

[5] Department of Chemistry, University of North Carolina, Chapel Hill, NC 27599-3290, USA

*Correspondence: ayers@mcmaster.ca (P.W.A.); shubin@email.unc.edu (S.L.)

Table S1. Linear regression equations between the S_0 polarizabilities (α_{iso}) and molecular volumes (Vol), molecular quadrupole moments (Θ_{iso}), and ITA quantities for all the systems in Schemes 1 and 2.

S_0	α_{iso}
S_S	$y = 0.9403x + 111.41$
I_F	$y = 0.0048x + 178.92$
S_{GBP}	$y = 0.2564x - 26.518$
${}^rR_2$	$y = 1.6976x - 26.078$
${}^rR_3$	$y = 1.6255x - 25.966$
I_G	$y = 115.99x + 20.233$
G_1	$y = -3.9841x + 41.292$
G_2	$y = 5.1989x + 62.811$
G_3	$y = 1.2932x - 3.9792$
Vol	$y = 0.0836x + 30.412$
Θ_{iso}	$y = -2.0683x - 15.249$

Table S2. Linear regression equations between the S_1 polarizabilities (α_{iso}) and molecular volumes (Vol), molecular quadrupole moments (Θ_{iso}), and ITA quantities for all the systems in Schemes 1 and 2.

S_1	α_{iso}
S_S	$y = 0.9888x + 105.09$
I_F	$y = 0.0031x + 188.9$
${}^rR_2$	$y = 1.6187x - 15.934$
${}^rR_3$	$y = 1.5517x - 16.217$
G_2	$y = 5.3296x + 58.111$
G_3	$y = 1.2826x - 1.087$
Vol	$y = 0.105x - 9.5127$
Θ_{iso}	$y = -1.9814x - 8.2371$

Table S3. Excited-state ITA quantities (in a.u.), molecular volume (Vol, in $Bohr^3/mol$), and molecular quadrupole moment (Θ_{iso}, in a.u.), based on the S_0 geometry with the transition density matrix as an input for all the systems in Schemes 1 and 2.

index	S_S	I_F	S_{GBP}	${}^rR_2$	${}^rR_3$	I_G	G_1	G_2	G_3	Vol	Θ_{iso}
1	96.62	4343.66	747.19	112.60	117.73	1.36	–35.10	23.95	139.60	1752.73	–91.17
2	*20.91*	*13967.03*	974.35	146.55	151.56	1.33	–34.68	23.61	141.54	2067.93	–111.17
3	143.16	5692.57	1017.29	154.00	161.88	2.08	–50.74	34.87	195.88	2360.41	–124.96
4	98.03	4793.24	802.00	120.73	126.10	1.43	–34.22	22.38	147.61	1746.57	–96.87
5	108.23	5045.39	855.88	129.04	135.06	1.58	–38.87	25.61	159.66	1968.24	–101.90
6	93.25	4917.75	802.25	120.55	125.53	1.33	–34.34	22.72	143.99	1682.49	–97.61
7	81.99	6523.98	855.67	128.54	133.50	1.32	–34.65	22.09	141.99	1893.44	–105.01
8	*20.86*	*13966.83*	974.31	146.55	151.55	1.33	–34.70	23.58	141.63	1949.76	–108.42
9	102.64	4928.22	829.73	124.72	130.02	1.42	–36.45	23.07	150.98	1961.87	–110.89
10	106.75	4595.15	801.16	120.89	126.54	1.51	–38.84	25.82	150.78	1959.19	–97.32
11	108.29	5045.38	855.91	128.99	134.82	1.56	–38.84	23.73	159.47	1869.73	–97.78
12	81.97	6524.04	855.65	128.58	133.67	1.34	–34.70	22.48	142.14	1804.34	–105.88
13	*20.92*	*13967.07*	974.34	146.59	151.71	1.35	–34.76	22.69	141.71	1920.89	–109.93
14	102.61	4928.35	829.69	124.75	130.11	1.44	–36.47	23.40	151.04	1982.69	–114.69
15	106.74	4595.21	801.16	120.89	126.62	1.51	–38.89	24.50	150.86	2011.10	–96.98
16	96.60	6318.28	966.13	144.77	150.18	1.45	–37.41	23.14	164.95	2068.91	–115.89
17	135.43	5825.99	1018.15	153.64	160.79	1.89	–52.49	36.56	192.01	2337.67	–124.54
18	135.35	5825.83	1018.08	153.64	160.85	1.89	–52.56	36.86	192.38	2494.60	–121.81
19	147.02	6527.75	1126.86	170.06	178.02	2.11	–56.25	38.42	212.03	2463.13	–132.50
20	153.40	7224.94	1222.48	184.28	192.65	2.23	–58.67	39.73	226.62	2664.72	–147.18
21	146.95	6527.57	1126.80	170.07	178.12	2.12	–56.26	39.01	212.40	2649.40	–129.04
22	153.33	7224.81	1222.41	184.28	192.70	2.23	–58.74	39.64	226.87	2985.18	–138.77
23	92.09	6775.55	909.62	136.86	142.49	1.49	–38.38	26.23	153.09	2148.93	–112.14
24	106.73	6569.76	1020.10	153.06	159.01	1.60	–41.10	26.28	176.00	2015.49	–121.95
25	174.15	7308.13	1289.03	194.68	203.90	2.43	–69.91	50.12	244.61	2925.68	–155.40
26	191.13	7329.96	1328.91	201.27	211.66	2.74	–74.36	52.65	255.14	3424.82	–165.98
27	83.02	6639.96	967.30	144.45	149.26	1.28	–32.81	20.06	158.31	1847.01	–115.66
28	96.94	4012.13	706.64	106.60	111.72	1.35	–37.28	25.87	130.48	1765.68	–83.35
29	92.07	4452.93	747.59	112.51	117.43	1.31	–35.58	24.40	137.22	1682.22	–87.76
30	80.88	5927.25	801.12	120.50	125.41	1.30	–36.23	25.64	133.52	1736.76	–94.74
31	90.33	4002.49	692.60	104.45	109.27	1.27	–35.88	24.86	129.30	1563.76	–83.12
32	103.87	4822.52	815.58	122.93	128.65	1.53	–41.03	28.13	147.55	1755.98	–95.54
33	128.46	5901.68	1004.89	151.69	158.91	1.93	–48.71	32.03	187.26	2350.61	–114.02
34	113.38	5647.72	937.82	141.10	147.14	1.62	–43.86	30.61	168.65	2239.20	–113.69
35	69.02	3451.38	571.43	85.89	89.61	0.99	–21.16	12.72	104.61	1341.88	–67.19
36	75.05	4146.01	667.18	100.00	103.77	1.07	–21.02	12.06	117.05	1405.54	–78.59

Table S4. Linear regression equations between the S_1 polarizabilities α_{iso}@S_1 and the S_0 polarizabilities α_{iso}@S_0, Vol@S_0, Θ_{iso}@S_0, and ITA quantities@S_0 for all the systems in Schemes 1 and 2.

	α_{iso}@S_1
α_{iso}@S_0	$y = 0.9729x + 4.9352$
S_S@S_0	$y = 0.9888x + 105.62$
I_F@S_0	$y = 0.0031x + 188.9$
S_{GBP}@S_0	$y = 0.2445x - 16.31$
${}^rR_2$@S_0	$y = 1.6202x - 16.071$
${}^rR_3$@S_0	$y = 1.5553x - 16.542$
I_G@S_0	$y = 115.7x + 19.988$
G_1@S_0	$y = -4.0048x + 39.69$
G_2@S_0	$y = 5.2401x + 60.918$
G_3@S_0	$y = 1.279x - 2.3708$
Vol@S_0	$y = 0.0865x + 23.472$
Θ_{iso}@S_0	$y = -1.9218x - 0.0716$

Table S5. Linear regression equations between the α_{iso}@S_0/S_1 and ITA quantities based on the transition density matrix for all the systems in Schemes 1 and 2.

	α_{iso}@S_0	α_{iso}@S_1
S_S	$y = 0.9404x + 111.18$	$y = 0.9886x + 105.41$
I_F	$y = 0.0048x + 178.92$	$y = 0.0031x + 188.9$
S_{GBP}	$y = 0.2564x – 26.625$	$y = 0.2445x – 16.402$
$^rR_2$	$y = 1.6974x – 25.972$	$y = 1.6201x – 15.981$
$^rR_3$	$y = 1.6247x – 25.688$	$y = 1.5547x – 16.293$
I_G	$y = 115.72x + 23.765$	$y = 115.65x + 23.141$
G_1	$y = –3.9604x + 43.674$	$y = –3.9741x + 42.372$
G_2	$y = 5.1788x + 64.117$	$y = 5.2107x + 62.494$
G_3	$y = 1.2918x – 3.4076$	$y = 1.2776x – 1.7986$
Vol	$y = 0.1028x – 4.2832$	$y = 0.1025x – 4.2863$
Θ_{iso}	$y = –2.1386x – 26.46$	$y = –1.9621x – 7.735$

Table S6. Molecular polarizabilities (α_{iso}, in Bohr^3/mol) at S_0/S_1 predicted by the original TS formula with conventional data as a reference for all the systems in Schemes 1 and 2

index	Ground-state (S_0)			Excited-state (S_1)		
	Becke	Hirshfeld	avg.	Becke	Hirshfeld	avg.
1	119.84	176.99	148.41	121.05	176.63	148.84
2	228.99	281.15	255.07	228.53	279.36	253.95
3	167.92	250.19	209.05	169.26	249.87	209.56
4	122.82	182.28	152.55	124.20	182.08	153.14
5	132.97	197.56	165.27	134.35	197.34	165.84
6	119.40	177.07	148.24	120.98	177.15	149.06
7	170.42	224.86	197.64	170.23	223.53	196.88
8	229.52	281.83	255.67	227.91	279.02	253.46
9	129.98	189.98	159.98	131.48	190.06	160.77
10	129.77	192.41	161.09	131.17	192.26	161.72
11	132.81	197.40	165.11	134.35	197.35	165.85
12	169.65	224.32	196.98	172.04	224.59	198.32
13	227.44	279.92	253.68	231.41	281.70	256.56
14	130.12	190.12	160.12	131.17	189.61	160.39
15	129.85	192.48	161.16	131.10	192.26	161.68
16	130.63	194.40	162.51	131.24	193.58	162.41
17	167.50	248.17	207.83	168.68	247.78	208.23
18	167.20	248.13	207.66	168.36	247.93	208.14
19	180.67	268.74	224.70	181.79	268.33	225.06
20	190.15	282.83	236.49	191.24	282.39	236.82
21	180.36	268.73	224.55	181.54	268.42	224.98
22	189.86	282.78	236.32	190.76	283.63	237.19
23	179.61	239.76	209.69	182.19	240.24	211.21
24	140.56	209.82	175.19	141.35	209.19	175.27
25	214.86	319.29	267.08	214.84	321.13	267.99
26	226.85	337.95	282.40	228.26	337.38	282.82
27	119.65	177.97	148.81	121.63	179.51	150.57
28	117.98	188.56	153.27	118.75	189.70	154.22
29	115.94	188.79	152.37	116.85	189.46	153.16
30	128.51	201.46	164.98	129.20	200.89	165.04
31	111.83	182.36	147.09	112.99	183.35	148.17
32	123.89	206.03	164.96	124.51	205.72	165.11
33	155.45	257.82	206.64	156.23	258.52	207.38
34	140.32	232.91	186.61	140.95	233.78	187.37
35	86.39	136.74	111.56	88.03	138.47	113.25
36	95.21	153.62	124.41	95.38	154.09	124.74

Table S7. Molecular polarizabilities (α_{iso}, in Bohr^3/mol) at S_0/S_1 predicted by the new TS formula with conventional data as a reference for all the systems in Schemes 1 and 2

index	Ground-state (S_0)			Excited-state (S_1)		
	Becke	Hirshfeld	avg.	Becke	Hirshfeld	avg.
1	119.84	176.99	148.41	121.05	176.63	148.84
2	228.99	281.15	255.07	228.53	279.36	253.95
3	167.92	250.19	209.05	169.26	249.87	209.56
4	122.82	182.28	152.55	124.20	182.08	153.14
5	132.97	197.56	165.27	134.35	197.34	165.84
6	119.40	177.07	148.24	120.98	177.15	149.06
7	170.42	224.86	197.64	170.23	223.53	196.88
8	229.52	281.83	255.67	227.91	279.02	253.46
9	129.98	189.98	159.98	131.48	190.06	160.77
10	129.77	192.41	161.09	131.17	192.26	161.72
11	132.81	197.40	165.11	134.35	197.35	165.85
12	169.65	224.32	196.98	172.04	224.59	198.32
13	227.44	279.92	253.68	231.41	281.70	256.56
14	130.12	190.12	160.12	131.17	189.61	160.39
15	129.85	192.48	161.16	131.10	192.26	161.68
16	130.63	194.40	162.51	131.24	193.58	162.41
17	167.50	248.17	207.83	168.68	247.78	208.23
18	167.20	248.13	207.66	168.36	247.93	208.14
19	180.67	268.74	224.70	181.79	268.33	225.06
20	190.15	282.83	236.49	191.24	282.39	236.82
21	180.36	268.73	224.55	181.54	268.42	224.98
22	189.86	282.78	236.32	190.76	283.63	237.19
23	179.61	239.76	209.69	182.19	240.24	211.21
24	140.56	209.82	175.19	141.35	209.19	175.27
25	214.86	319.29	267.08	214.84	321.13	267.99
26	226.85	337.95	282.40	228.26	337.38	282.82
27	119.65	177.97	148.81	121.63	179.51	150.57
28	100.17	183.02	141.59	101.03	184.50	142.76
29	98.36	184.42	141.39	99.36	185.29	142.32
30	110.44	196.70	153.57	111.04	196.11	153.58
31	94.34	177.54	135.94	95.61	178.80	137.20
32	103.91	200.37	152.14	104.49	199.99	152.24
33	131.32	251.24	191.28	132.17	252.12	192.15
34	118.91	227.28	173.09	119.56	228.50	174.03
35	74.15	133.30	103.72	76.08	135.55	105.81
36	81.89	150.39	116.14	81.99	151.00	116.49

Cartesian coordinates optimized at the CAM-B3LYP/6-311+G(d) level

26			
**1**			
C	1.85598	0.58956	-0.00015
C	3.22436	-1.39818	0.00039
C	4.34370	-0.63354	0.00024
C	4.24113	0.78375	-0.00011
C	3.01965	1.38770	-0.00030
C	-0.13442	-0.25313	-0.00001
C	0.72884	-1.32570	0.00029
H	3.22658	-2.47964	0.00065
H	5.31191	-1.11632	0.00039
H	5.14365	1.38262	-0.00023
H	2.90424	2.46360	-0.00057
H	0.56850	-2.38996	0.00059
N	1.99656	-0.79204	0.00020
N	0.57604	0.91819	-0.00027
C	-1.59991	-0.25338	-0.00005
C	-2.31178	-1.45782	-0.00011
C	-2.32937	0.95069	-0.00002
C	-3.69174	-1.49056	-0.00010
H	-1.76322	-2.39371	-0.00018
C	-3.72485	0.91407	-0.00001
C	-4.39904	-0.28972	-0.00004
H	-4.21526	-2.43927	-0.00014
H	-4.25321	1.86009	0.00003
H	-5.48357	-0.29658	-0.00003
O	-1.75109	2.16494	0.00000
H	-0.77442	2.06413	-0.00013

26			
**2**			
C	-3.36093	0.56690	0.00001
C	-4.67708	-1.45576	0.00003
C	-5.81550	-0.72027	0.00003
C	-5.74970	0.69929	0.00001
C	-4.54468	1.33504	-0.00000
C	-1.35009	-0.22431	0.00002
C	-2.18433	-1.31887	0.00003
H	-4.65107	-2.53687	0.00005
H	-6.77094	-1.22778	0.00003
H	-6.66753	1.27437	0.00000
H	-4.45737	2.41355	-0.00002

H	-1.99681	-2.37875	0.00003
N	-3.46532	-0.81746	0.00003
N	-2.08960	0.92849	-0.00000
C	0.11423	-0.18263	0.00002
C	0.86416	-1.36135	0.00000
C	0.80757	1.04296	0.00002
C	2.24474	-1.36019	-0.00002
H	0.34941	-2.31584	0.00000
C	2.20401	1.05467	-0.00000
C	2.89941	-0.13368	-0.00002
H	2.80242	-2.28698	-0.00003
O	0.19635	2.23707	0.00005
H	2.70863	2.01170	0.00000
Br	4.80185	-0.09673	-0.00004
H	-0.77851	2.10762	0.00009

40			
**3**			
C	-3.53893	0.55033	0.00009
C	-4.81954	-1.49493	-0.00026
C	-5.97122	-0.77976	-0.00014
C	-5.93035	0.64056	0.00011
C	-4.73622	1.29706	0.00022
C	-1.51354	-0.20490	-0.00004
C	-2.32939	-1.31409	-0.00023
H	-4.77476	-2.57547	-0.00045
H	-6.91751	-1.30421	-0.00022
H	-6.85805	1.19966	0.00020
H	-4.66772	2.37696	0.00040
H	-2.12277	-2.37037	-0.00041
N	-3.61934	-0.83607	-0.00015
N	-2.27447	0.93444	0.00015
C	-0.05031	-0.14008	-0.00003
C	0.71876	-1.30901	-0.00027
C	0.62797	1.09220	0.00022
C	2.09730	-1.27936	-0.00026
H	0.21600	-2.27047	-0.00048
C	2.02400	1.11661	0.00023
C	2.76325	-0.04856	-0.00001
H	2.65787	-2.20687	-0.00046
H	2.52735	2.07516	0.00043
O	-0.00060	2.28098	0.00047
N	4.19888	0.03852	0.00002
C	4.81062	-0.50513	-1.21243

H	5.89217	-0.39699	-1.09561
H	4.61690	-1.58684	-1.31601
C	4.36863	0.21748	-2.47337
H	4.58206	1.28561	-2.40031
H	4.89939	-0.18083	-3.34132
H	3.29911	0.10053	-2.65466
C	4.81062	-0.50572	1.21219
H	4.61691	-1.58749	1.31525
H	5.89217	-0.39752	1.09543
C	4.36863	0.21627	2.47349
H	4.58207	1.28443	2.40096
H	3.29910	0.09924	2.65471
H	4.89936	-0.18248	3.34125
H	-0.97187	2.13815	0.00043

27<br>**4**			
C	2.25803	0.58123	-0.00003
C	3.60812	-1.41782	0.00003
C	4.73498	-0.66335	-0.00001
C	4.64499	0.75411	-0.00005
C	3.42827	1.36875	-0.00006
C	0.25890	-0.24415	0.00001
C	1.11325	-1.32425	0.00004
H	3.60077	-2.49927	0.00006
H	5.69867	-1.15510	0.00000
H	5.55256	1.34533	-0.00008
H	3.32258	2.44566	-0.00010
H	0.94379	-2.38715	0.00009
N	2.38624	-0.80131	0.00001
N	0.98044	0.92101	-0.00003
C	-1.20359	-0.22673	0.00001
C	-1.94010	-1.41633	0.00004
C	-1.92055	0.98490	-0.00002
C	-3.31726	-1.43738	0.00005
H	-1.41008	-2.36267	0.00006
C	-3.31482	0.97229	-0.00001
C	-4.00496	-0.22471	0.00003
H	-3.87383	-2.36576	0.00008
O	-1.32915	2.19045	-0.00006
H	-3.83086	1.92712	-0.00003
O	-5.36493	-0.27905	0.00004
H	-5.73691	0.60892	0.00003

H	-0.35243	2.07460	-0.00006

30			
**5**			
C	-2.63606	0.61229	-0.00014
C	-4.05484	-1.33874	0.00009
C	-5.15496	-0.54568	-0.00001
C	-5.01582	0.86778	-0.00018
C	-3.77844	1.43979	-0.00024
C	-0.66664	-0.28191	-0.00003
C	-1.55820	-1.33178	0.00010
H	-4.08505	-2.41981	0.00022
H	-6.13512	-1.00373	0.00005
H	-5.90232	1.49017	-0.00026
H	-3.63542	2.51239	-0.00038
H	-1.42557	-2.39989	0.00026
N	-2.81234	-0.76513	0.00003
N	-1.34761	0.90764	-0.00017
C	0.79500	-0.31623	-0.00002
C	1.49014	-1.53324	-0.00004
C	1.55596	0.86438	0.00003
C	2.86247	-1.59941	0.00000
H	0.92802	-2.46095	-0.00010
C	2.95252	0.80952	0.00008
C	3.60206	-0.41106	0.00006
H	3.38747	-2.54618	-0.00002
H	3.48109	1.75251	0.00011
O	1.00956	2.09221	0.00004
O	4.95085	-0.55433	0.00010
C	5.75124	0.60889	0.00018
H	5.57213	1.21519	0.89258
H	6.78258	0.26393	0.00022
H	5.57221	1.21525	-0.89219
H	0.02969	2.01283	-0.00005

26			
**6**			
C	-2.21599	0.54922	0.00013
C	-3.37670	-1.56717	-0.00015
C	-4.56699	-0.91928	-0.00003
C	-4.60811	0.50146	0.00018
C	-3.45449	1.22604	0.00026
C	-0.15247	-0.08828	-0.00000
C	-0.90152	-1.24273	-0.00015

H	-3.26940	-2.64326	-0.00031
H	-5.48163	-1.49710	-0.00010
H	-5.56666	1.00575	0.00027
H	-3.44849	2.30806	0.00042
H	-0.63412	-2.28539	-0.00031
N	-2.21627	-0.83938	-0.00007
N	-0.97589	1.00581	0.00018
C	1.30530	0.06024	-0.00002
C	2.12803	-1.07227	-0.00020
C	1.90972	1.33060	0.00015
C	3.49213	-0.93530	-0.00022
H	1.70534	-2.06965	-0.00033
C	3.30198	1.43267	0.00012
C	4.10117	0.30720	-0.00006
H	3.73541	2.42529	0.00025
H	5.18188	0.38013	-0.00008
O	1.21407	2.48309	0.00034
F	4.26506	-2.04825	-0.00040
H	0.25235	2.28633	0.00035

26			
7			
C	2.57735	0.47242	0.00009
C	3.52470	-1.74744	-0.00006
C	4.77279	-1.21941	0.00002
C	4.95306	0.19038	0.00013
C	3.87609	1.02466	0.00017
C	0.46104	0.03998	0.00000
C	1.09329	-1.18217	-0.00007
H	3.31230	-2.80779	-0.00014
H	5.62634	-1.88415	-0.00001
H	5.95644	0.59823	0.00019
H	3.97632	2.10204	0.00026
H	0.72549	-2.19378	-0.00016
N	2.44129	-0.90930	-0.00002
N	1.38770	1.04818	0.00011
C	-0.97501	0.33342	-0.00001
C	-1.90668	-0.70913	-0.00006
C	-1.44857	1.65850	0.00003
C	-3.25893	-0.45103	-0.00007
H	-1.56813	-1.73778	-0.00010
C	-2.82375	1.89731	0.00003
C	-3.72884	0.85664	-0.00003
H	-3.15845	2.92754	0.00006

H	-4.79390	1.05230	-0.00003
O	-0.64193	2.73262	0.00007
Cl	-4.39642	-1.78320	-0.00014
H	0.29606	2.43994	0.00007

26			
**8**			
C	2.88003	-0.71195	0.00021
C	4.69066	0.88305	0.00062
C	5.58958	-0.13114	0.00059
C	5.14507	-1.48101	0.00037
C	3.81372	-1.76997	0.00018
C	1.15501	0.59012	0.00019
C	2.25271	1.42030	0.00041
H	4.95587	1.93143	0.00078
H	6.64622	0.10111	0.00073
H	5.87484	-2.28133	0.00034
H	3.43980	-2.78533	0.00001
H	2.35708	2.49147	0.00056
N	3.35189	0.59345	0.00043
N	1.55840	-0.71830	0.00007
C	-0.26736	0.94907	0.00008
C	-0.65748	2.28943	0.00020
C	-1.26101	-0.05217	-0.00014
C	-1.98812	2.65707	0.00010
H	0.10367	3.06150	0.00037
C	-2.60509	0.34065	-0.00024
C	-2.96894	1.67195	-0.00012
H	-2.26947	3.70311	0.00019
H	-4.01879	1.93697	-0.00020
O	-0.98352	-1.35916	-0.00026
Br	-3.95024	-0.99588	-0.00054
H	-0.00741	-1.48821	-0.00016

27			
**9**			
C	2.44898	0.46707	0.00004
C	3.42787	-1.73884	-0.00010
C	4.66779	-1.19260	-0.00006
C	4.82768	0.21971	0.00004
C	3.73925	1.03868	0.00009
C	0.33946	0.00290	0.00001
C	0.98865	-1.20938	-0.00008
H	3.23049	-2.80204	-0.00017

H	5.53093	-1.84475	-0.00010
H	5.82512	0.64179	0.00007
H	3.82422	2.11734	0.00016
H	0.63612	-2.22649	-0.00015
N	2.33248	-0.91575	-0.00005
N	1.25027	1.02507	0.00008
C	-1.10062	0.27901	0.00002
C	-2.02263	-0.76328	-0.00006
C	-1.58791	1.60339	0.00011
C	-3.39117	-0.52822	-0.00006
H	-1.67234	-1.78853	-0.00012
C	-2.96664	1.83622	0.00010
C	-3.85998	0.79119	0.00002
H	-3.30446	2.86517	0.00017
H	-4.92600	0.98355	0.00002
O	-0.79075	2.67537	0.00020
C	-4.30617	-1.62605	-0.00014
N	-5.03956	-2.51168	-0.00021
H	0.15229	2.38876	0.00021

29			
**10**			
C	-2.24173	0.53292	0.00003
C	-3.36354	-1.60377	-0.00003
C	-4.56617	-0.97810	0.00000
C	-4.63325	0.44129	0.00005
C	-3.49238	1.18649	0.00006
C	-0.16491	-0.06640	-0.00003
C	-0.89440	-1.23429	-0.00003
H	-3.23690	-2.67781	-0.00006
H	-5.46991	-1.57289	-0.00000
H	-5.60071	0.92837	0.00007
H	-3.50602	2.26848	0.00009
H	-0.60836	-2.27183	-0.00005
N	-2.21677	-0.85574	-0.00002
N	-1.01030	1.01191	0.00002
C	1.28970	0.10911	-0.00003
C	2.13993	-1.00486	-0.00001
C	1.86805	1.38853	-0.00003
C	3.51740	-0.89766	0.00002
H	1.69757	-1.99637	-0.00000
C	3.25903	1.50830	-0.00001
C	4.06416	0.39021	0.00002
H	3.67984	2.50695	-0.00002

H	5.14318	0.51362	0.00003
O	1.15397	2.53014	-0.00010
C	4.40639	-2.11249	0.00008
H	3.82233	-3.03447	-0.00020
H	5.05558	-2.13503	-0.87929
H	5.05512	-2.13528	0.87978
H	0.19628	2.31628	-0.00019

30			
**11**			
C	-2.63839	0.46038	-0.00003
C	-3.60464	-1.75171	-0.00003
C	-4.84858	-1.21347	-0.00005
C	-5.01711	0.19779	-0.00006
C	-3.93287	1.02280	-0.00005
C	-0.52563	0.01087	0.00001
C	-1.16860	-1.20614	-0.00001
H	-3.40130	-2.81388	-0.00003
H	-5.70757	-1.87124	-0.00007
H	-6.01704	0.61416	-0.00007
H	-4.02394	2.10104	-0.00005
H	-0.80758	-2.22012	-0.00001
N	-2.51418	-0.92322	-0.00002
N	-1.44466	1.02636	-0.00001
C	0.91436	0.28720	0.00002
C	1.82862	-0.76563	0.00002
C	1.40446	1.60745	0.00002
C	3.19501	-0.54728	0.00001
H	1.48581	-1.79370	0.00003
C	2.77802	1.81707	0.00001
C	3.67362	0.76026	0.00000
H	3.13301	2.84080	0.00001
H	4.73493	0.97101	-0.00001
O	0.61004	2.69934	0.00006
O	3.98416	-1.66472	0.00000
C	5.38157	-1.48864	0.00006
H	5.81286	-2.48735	0.00009
H	5.71960	-0.95235	-0.89241
H	5.71953	-0.95233	0.89255
H	-0.32912	2.41922	0.00011

26			
**12**			
C	-1.02496	0.86945	0.00024

C	-2.57142	-0.98680	-0.00036
C	-3.60611	-0.11157	-0.00017
C	-3.38411	1.28978	0.00023
C	-2.10987	1.77034	0.00044
C	0.87694	-0.15487	0.00008
C	-0.08416	-1.14288	-0.00028
H	-2.68910	-2.06076	-0.00067
H	-4.23390	1.95962	0.00037
H	-1.89904	2.83147	0.00075
H	-0.02446	-2.21741	-0.00064
N	-1.29478	-0.49293	-0.00015
N	0.27945	1.07673	0.00037
C	2.33519	-0.29485	0.00013
C	2.92801	-1.56249	0.00019
C	3.17662	0.83367	0.00007
C	4.29813	-1.72719	0.00014
H	2.29267	-2.44174	0.00028
C	4.56211	0.66335	0.00002
C	5.11734	-0.59950	0.00005
H	4.72836	-2.72160	0.00019
H	5.17881	1.55425	-0.00003
H	6.19613	-0.71049	0.00001
O	2.71854	2.09824	0.00008
Cl	-5.23590	-0.71992	-0.00044
H	1.73778	2.09416	0.00031

26			
**13**			
C	0.29386	0.98681	0.00007
C	1.91774	-0.80229	-0.00004
C	2.91561	0.11487	0.00001
C	2.63367	1.50625	0.00010
C	1.34002	1.93251	0.00013
C	-1.56304	-0.11733	0.00001
C	-0.56132	-1.06360	-0.00004
H	2.07505	-1.87114	-0.00010
H	3.45107	2.21501	0.00014
H	1.08471	2.98390	0.00019
H	-0.57543	-2.13970	-0.00011
N	0.62111	-0.36286	-0.00001
N	-1.01780	1.13884	0.00008
C	-3.01414	-0.31814	0.00000
C	-3.55338	-1.60955	0.00007
C	-3.90209	0.77414	-0.00006

C	-4.91534	-1.83157	0.00008
H	-2.88166	-2.46131	0.00013
C	-5.27927	0.54578	-0.00005
C	-5.78108	-0.73918	0.00001
H	-5.30360	-2.84311	0.00013
H	-5.93276	1.41004	-0.00010
H	-6.85428	-0.89536	0.00002
O	-3.49758	2.05684	-0.00015
Br	4.71498	-0.47575	-0.00003
H	-2.51746	2.09430	-0.00017

27<br>**14**			
C	1.16629	0.85916	-0.00002
C	2.70292	-1.00083	-0.00009
C	3.75457	-0.13165	-0.00010
C	3.52358	1.27745	-0.00007
C	2.25286	1.76026	-0.00003
C	-0.73639	-0.16727	0.00001
C	0.22005	-1.15646	-0.00004
H	2.81630	-2.07595	-0.00011
H	4.37253	1.94853	-0.00008
H	2.04126	2.82112	-0.00001
H	0.15778	-2.23080	-0.00006
N	1.43521	-0.50938	-0.00006
N	-0.13304	1.06613	0.00001
C	-2.19399	-0.30106	0.00004
C	-2.78942	-1.56831	-0.00004
C	-3.03284	0.82943	0.00015
C	-4.15913	-1.72999	-0.00002
H	-2.15626	-2.44904	-0.00013
C	-4.41881	0.66144	0.00017
C	-4.97596	-0.60003	0.00009
H	-4.59162	-2.72329	-0.00009
H	-5.03379	1.55343	0.00026
H	-6.05489	-0.70893	0.00010
O	-2.57511	2.09385	0.00026
C	5.08662	-0.64821	-0.00014
N	6.16113	-1.05421	-0.00017
H	-1.59558	2.09287	0.00026

29<br>**15**			
C	1.41013	0.77135	0.00003

C	2.89940	-1.12847	0.00004
C	3.98555	-0.31482	0.00007
C	3.77894	1.09787	0.00009
C	2.52770	1.63286	0.00007
C	-0.52513	-0.18827	-0.00003
C	0.40355	-1.20573	0.00000
H	2.95856	-2.20895	0.00003
H	4.64445	1.75143	0.00011
H	2.35517	2.70118	0.00008
H	0.30917	-2.27790	0.00000
N	1.63511	-0.59469	0.00002
N	0.11189	1.02376	0.00001
C	-1.98807	-0.27906	-0.00005
C	-2.62493	-1.52459	0.00002
C	-2.79006	0.87793	-0.00010
C	-4.00053	-1.64178	0.00006
H	-2.02007	-2.42515	0.00006
C	-4.18060	0.75617	-0.00006
C	-4.78009	-0.48670	0.00002
H	-4.46487	-2.62085	0.00012
H	-4.76585	1.66813	-0.00011
H	-5.86219	-0.55990	0.00004
O	-2.28643	2.12520	-0.00025
C	5.37860	-0.87623	0.00010
H	5.93631	-0.54621	-0.87969
H	5.93626	-0.54627	0.87993
H	5.37387	-1.96693	0.00006
H	-1.30521	2.08326	-0.00037

29			
**16**			
C	0.36293	0.97468	0.00014
C	1.96916	-0.82652	-0.00028
C	2.97950	0.07834	-0.00017
C	2.70329	1.47503	0.00010
C	1.41685	1.91466	0.00025
C	-1.50145	-0.11914	0.00004
C	-0.50909	-1.07230	-0.00019
H	2.11916	-1.89647	-0.00049
H	3.52552	2.17962	0.00017
H	1.16768	2.96733	0.00046
H	-0.53155	-2.14826	-0.00039
N	0.68082	-0.38059	-0.00013
N	-0.94413	1.13510	0.00024

C	-2.95382	-0.30651	0.00008
C	-3.50305	-1.59407	-0.00014
C	-3.83305	0.79277	0.00035
C	-4.86634	-1.80534	-0.00011
H	-2.83825	-2.45120	-0.00035
C	-5.21200	0.57496	0.00038
C	-5.72351	-0.70593	0.00016
H	-5.26248	-2.81375	-0.00028
H	-5.85879	1.44420	0.00059
H	-6.79785	-0.85367	0.00019
O	-3.42031	2.07279	0.00057
C	4.40043	-0.38569	-0.00027
F	5.06870	0.06481	-1.07802
F	4.50104	-1.72478	-0.00096
F	5.06848	0.06366	1.07812
H	-2.44085	2.10552	0.00052

36			
**17**			
C	-0.23230	1.08470	-0.13534
C	1.48195	-0.60242	0.06319
C	2.45970	0.33746	-0.05411
C	2.07682	1.70449	-0.21338
C	0.76717	2.07254	-0.25474
C	-2.03017	-0.10397	0.01558
C	-0.98111	-0.99019	0.11794
H	1.67960	-1.65472	0.21532
H	2.85161	2.45317	-0.32535
H	0.46119	3.10217	-0.38576
H	-0.93903	-2.05782	0.24723
N	0.16429	-0.23477	0.01931
N	-1.55115	1.17010	-0.13994
C	-3.46965	-0.37561	0.05425
C	-3.94393	-1.68274	0.20922
C	-4.41136	0.66433	-0.06199
C	-5.29344	-1.97000	0.25036
H	-3.23013	-2.49458	0.29967
C	-5.77539	0.37045	-0.01987
C	-6.21274	-0.92905	0.13424
H	-5.63044	-2.99270	0.37144
H	-6.47125	1.19589	-0.11225
H	-7.27685	-1.13648	0.16448
O	-4.07006	1.95631	-0.21479
C	3.88820	-0.05495	-0.01132

C	4.34870	-1.16839	-0.71455
C	4.80318	0.68496	0.73879
C	5.68465	-1.53911	-0.66031
H	3.65946	-1.73474	-1.33149
C	6.13929	0.31681	0.79056
H	4.46182	1.54443	1.30541
C	6.58466	-0.79787	0.09261
H	6.02529	-2.40388	-1.21880
H	6.83425	0.89948	1.38493
H	7.62929	-1.08505	0.13251
H	-3.09171	2.03846	-0.23141

36			
**18**			
C	0.65407	1.05443	0.04337
C	0.81497	3.46777	0.06609
C	2.16610	3.37619	0.07249
C	2.79573	2.10621	0.06882
C	2.07311	0.94210	0.05375
C	-1.49273	0.77767	0.00217
C	-1.28873	2.13749	0.02438
H	0.26257	4.39716	0.07342
H	2.75864	4.28129	0.09068
H	3.87728	2.05636	0.10155
H	-1.96273	2.97661	0.01413
N	0.07479	2.31656	0.05122
N	-0.28797	0.12684	0.01342
C	-2.75541	0.03478	-0.03274
C	-3.98002	0.70855	0.02668
C	-2.77237	-1.36947	-0.13011
C	-5.18562	0.03713	-0.01079
H	-3.98191	1.79032	0.10828
C	-3.99318	-2.04538	-0.16779
C	-5.18529	-1.35293	-0.10956
H	-6.11830	0.58655	0.03802
H	-3.96818	-3.12597	-0.24423
H	-6.12313	-1.89684	-0.13977
O	-1.65752	-2.11999	-0.19070
C	2.72880	-0.38530	0.04214
C	3.84342	-0.60544	-0.76799
C	2.27115	-1.42759	0.84952
C	4.49099	-1.83202	-0.76555
H	4.19240	0.18430	-1.42418
C	2.92170	-2.65196	0.85427

H	1.40375	-1.27673	1.47911
C	4.03301	-2.85902	0.04788
H	5.34988	-1.98806	-1.40887
H	2.55498	-3.44990	1.49012
H	4.53564	-3.81976	0.04840
H	-0.86670	-1.53852	-0.15569

40			
**19**			
C	-1.08927	1.13335	-0.15434
C	0.67801	-0.50306	0.00075
C	1.62649	0.46515	-0.12839
C	1.19872	1.82107	-0.27166
C	-0.12161	2.15104	-0.28418
C	-2.84829	-0.10905	0.01757
C	-1.77152	-0.96440	0.09328
H	0.90812	-1.55016	0.14287
H	1.94850	2.59310	-0.39524
H	-0.46002	3.17212	-0.40194
H	-1.69532	-2.03132	0.21264
N	-0.65121	-0.17426	-0.01876
N	-2.41046	1.17983	-0.13487
C	-4.27825	-0.42382	0.07998
C	-4.71103	-1.74605	0.22731
C	-5.25213	0.58917	-0.00527
C	-6.05061	-2.07349	0.29085
H	-3.97207	-2.53749	0.29333
C	-6.60588	0.25480	0.05946
C	-7.00206	-1.05882	0.20565
H	-6.35507	-3.10712	0.40523
H	-7.32724	1.06045	-0.00924
H	-8.05888	-1.29785	0.25371
O	-4.95136	1.89233	-0.14844
C	3.06551	0.11601	-0.11737
C	3.55100	-0.98824	-0.82582
C	3.98006	0.87582	0.60318
C	4.88982	-1.32180	-0.80357
H	2.86975	-1.58162	-1.42601
C	5.33231	0.55490	0.63356
H	3.63524	1.72918	1.17707
C	5.79287	-0.55167	-0.07160
H	5.26591	-2.17186	-1.36023
H	6.00677	1.16933	1.21477
O	7.08748	-0.95464	-0.11175

C	8.05029	-0.20620	0.60054
H	7.83874	-0.20264	1.67369
H	9.00348	-0.69885	0.42517
H	8.10418	0.82362	0.23585
H	-3.97606	2.00246	-0.18431

42			
**20**			
C	1.81114	1.17026	0.19164
C	-0.00338	-0.40948	0.00316
C	-0.92037	0.58467	0.16175
C	-0.45464	1.92356	0.33833
C	0.87511	2.21232	0.35599
C	3.53117	-0.11856	-0.02875
C	2.42964	-0.93958	-0.12261
H	-0.26659	-1.44498	-0.16425
H	-1.18019	2.71438	0.48452
H	1.24470	3.21895	0.50067
H	2.32172	-1.99993	-0.27195
N	1.33335	-0.12090	0.02025
N	3.13163	1.17795	0.16466
C	4.95049	-0.47292	-0.10955
C	5.34223	-1.80211	-0.30393
C	5.95479	0.50690	0.00475
C	6.67072	-2.16731	-0.38509
H	4.57956	-2.56830	-0.39349
C	7.29753	0.13406	-0.07780
C	7.65282	-1.18508	-0.27019
H	6.94338	-3.20505	-0.53609
H	8.04329	0.91470	0.01441
H	8.70165	-1.45417	-0.33152
O	5.69527	1.81313	0.19314
C	-2.36875	0.27691	0.14241
C	-2.87579	-0.83907	0.81110
C	-3.25798	1.10009	-0.55064
C	-4.22811	-1.13020	0.77747
H	-2.20757	-1.47029	1.38600
C	-4.61230	0.81420	-0.58334
H	-2.88264	1.96198	-1.09064
C	-5.10559	-0.30586	0.08014
H	-4.62403	-1.99211	1.30037
H	-5.29117	1.45480	-1.13082
C	-6.54941	-0.66147	0.07837
O	-7.01305	-1.62196	0.63812

O	-7.29455	0.20736	-0.62024
C	-8.69567	-0.07153	-0.66767
H	-9.13252	0.72307	-1.26612
H	-8.87486	-1.04176	-1.12930
H	-9.11762	-0.07128	0.33656
H	4.72475	1.95360	0.23586

40<br>**21**			
C	-0.29160	1.44992	0.04634
C	-0.84352	3.80482	0.00760
C	0.47519	4.11156	0.03283
C	1.44815	3.08155	0.07330
C	1.09895	1.75594	0.07848
C	-2.26288	0.55637	-0.01502
C	-2.46518	1.91653	-0.02460
H	-1.64346	4.53171	-0.01683
H	0.77746	5.15051	0.03420
H	2.49584	3.35170	0.12514
H	-3.35444	2.52156	-0.06576
N	-1.21420	2.48687	0.01523
N	-0.92114	0.28658	0.02787
C	-3.25317	-0.52343	-0.04854
C	-4.62179	-0.23631	-0.01124
C	-2.85798	-1.87243	-0.12338
C	-5.57818	-1.23118	-0.04974
H	-4.94051	0.79862	0.05393
C	-3.82746	-2.87605	-0.16214
C	-5.17058	-2.56156	-0.12649
H	-6.63125	-0.97774	-0.01819
H	-3.48680	-3.90302	-0.22079
H	-5.90816	-3.35614	-0.15685
O	-1.57177	-2.26470	-0.16149
C	2.11029	0.67818	0.10614
C	3.26320	0.77523	-0.66464
C	1.95863	-0.45210	0.91851
C	4.24775	-0.20579	-0.63326
H	3.39426	1.62342	-1.32767
C	2.92948	-1.42974	0.96432
H	1.06892	-0.56090	1.52509
C	4.08287	-1.31515	0.18802
H	5.12306	-0.09524	-1.25922
H	2.81475	-2.30232	1.59609
O	4.97625	-2.33032	0.29707

C	6.15533	-2.27788	-0.47700
H	6.71309	-3.17977	-0.23668
H	5.92967	-2.26839	-1.54731
H	6.76027	-1.40202	-0.22443
H	-0.98611	-1.47698	-0.12408

42			
**22**			
C	-1.06596	1.55370	0.04851
C	-2.00557	3.78242	0.04880
C	-0.75763	4.30844	0.07310
C	0.37503	3.45687	0.08884
C	0.25105	2.09219	0.07797
C	-2.85875	0.34252	-0.01885
C	-3.28726	1.64929	-0.00782
H	-2.91637	4.36502	0.04043
H	-0.63532	5.38323	0.09021
H	1.36305	3.89832	0.13553
H	-4.26605	2.09598	-0.03724
N	-2.15024	2.42195	0.03552
N	-1.48990	0.30219	0.01526
C	-3.65410	-0.88710	-0.06482
C	-5.05119	-0.83322	-0.01036
C	-3.04009	-2.14936	-0.16973
C	-5.82815	-1.97290	-0.06125
H	-5.53767	0.13230	0.07899
C	-3.82877	-3.30002	-0.22112
C	-5.20483	-3.21485	-0.16839
H	-6.90827	-1.89986	-0.01566
H	-3.32212	-4.25430	-0.30287
H	-5.79970	-4.12078	-0.20871
O	-1.70711	-2.32128	-0.22591
C	1.43389	1.20261	0.09434
C	2.54519	1.50685	-0.69369
C	1.47844	0.06876	0.90802
C	3.67696	0.70991	-0.66436
H	2.51417	2.36518	-1.35514
C	2.61059	-0.72533	0.94376
H	0.62337	-0.18786	1.51936
C	3.71677	-0.41088	0.15961
H	4.52950	0.95015	-1.28591
H	2.65148	-1.60105	1.57955
C	4.90651	-1.29919	0.23518
O	4.99138	-2.26554	0.94873

O	5.89582	-0.90944	-0.58439
C	7.07185	-1.72029	-0.56687
H	6.83441	-2.74177	-0.86163
H	7.75269	-1.26516	-1.28099
H	7.51251	-1.73099	0.42938
H	-1.25930	-1.44968	-0.17358

29			
**23**			
C	1.41461	0.94381	0.00001
C	2.78360	-1.04704	0.00011
C	3.89459	-0.27086	0.00011
C	3.80268	1.14490	0.00006
C	2.57804	1.74074	0.00001
C	-0.57372	0.09911	-0.00002
C	0.29253	-0.97327	0.00004
H	2.80192	-2.12728	0.00014
H	4.71054	1.73364	0.00007
H	2.46587	2.81679	-0.00003
H	0.13408	-2.03769	0.00007
N	1.55796	-0.43780	0.00006
N	0.13502	1.27050	-0.00003
C	-2.03831	0.09504	-0.00006
C	-2.74475	-1.11564	-0.00006
C	-2.76955	1.29349	-0.00011
C	-4.12461	-1.17870	-0.00010
H	-2.18409	-2.04533	-0.00003
C	-4.16468	1.24108	-0.00016
C	-4.82561	0.03243	-0.00016
H	-4.70526	2.18025	-0.00022
H	-5.91155	0.02198	-0.00022
O	-2.20253	2.51485	-0.00012
Cl	5.46146	-1.02686	0.00018
C	-4.85771	-2.49336	-0.00000
H	-4.16515	-3.33682	-0.00086
H	-5.49822	-2.59549	0.88000
H	-5.49963	-2.59484	-0.87905
H	-1.22664	2.42345	-0.00009

32			
**24**			
C	-0.74480	-1.10391	0.00004
C	-2.20381	0.81854	-0.00009
C	-3.28266	-0.00360	-0.00007

C	-3.11764	-1.41771	0.00001
C	-1.86985	-1.95752	0.00006
C	1.20034	-0.16081	0.00003
C	0.28610	0.86779	-0.00006
H	-2.26891	1.89699	-0.00015
H	-3.99293	-2.05519	0.00001
H	-1.70464	-3.02660	0.00012
H	0.39348	1.93857	-0.00013
N	-0.95484	0.27242	-0.00004
N	0.54530	-1.36722	0.00008
C	2.66261	-0.08978	0.00004
C	3.31114	1.15337	0.00003
C	3.44941	-1.25250	0.00005
C	4.68608	1.28151	0.00003
H	2.70738	2.05563	0.00004
C	4.84064	-1.13396	0.00005
C	5.44341	0.10440	0.00005
H	5.42500	-2.04648	0.00008
H	6.52760	0.16613	0.00006
O	2.94259	-2.49998	0.00009
C	-4.66228	0.57137	-0.00006
F	-5.36442	0.17550	-1.07785
F	-4.65679	1.91434	-0.00054
F	-5.36408	0.17629	1.07825
C	5.35670	2.62901	-0.00005
H	4.62554	3.43917	0.00067
H	5.99305	2.76020	0.87906
H	5.99184	2.76069	-0.87997
H	1.96402	-2.45802	0.00014

46<br>**25**			
C	0.24166	0.04452	0.02300
C	-1.46386	-1.67029	-0.01402
C	-2.44335	-0.72732	0.01234
C	-2.06629	0.64658	0.04450
C	-0.75968	1.05464	0.05204
C	2.04189	-1.15471	-0.04201
C	0.99384	-2.04557	-0.05002
H	-1.65147	-2.73393	-0.06408
H	-2.84873	1.39268	0.11150
H	0.94986	-3.12023	-0.09077
N	-0.15134	-1.28483	-0.00547
N	1.56156	0.12689	0.00217

C	3.48180	-1.42419	-0.07689
C	3.96142	-2.73816	-0.05200
C	4.41830	-0.37498	-0.13936
C	5.31152	-3.02372	-0.09050
H	3.25129	-3.55648	0.00238
C	5.78273	-0.66711	-0.17832
C	6.22560	-1.97380	-0.15457
H	5.65294	-4.05189	-0.06906
H	6.47463	0.16534	-0.22763
H	7.29002	-2.17966	-0.18500
O	4.07157	0.92470	-0.16521
C	-0.39054	2.48805	0.09217
C	-1.08810	3.41081	-0.68824
C	0.63424	2.94979	0.91936
C	-0.77783	4.76166	-0.63725
H	-1.86617	3.06417	-1.35944
C	0.94090	4.30089	0.97280
H	1.19099	2.24737	1.52606
C	0.23705	5.21135	0.19582
H	-1.32451	5.46303	-1.25782
H	1.73766	4.64299	1.62366
H	0.48361	6.26652	0.23441
C	-3.87120	-1.12509	0.01284
C	-4.32515	-2.17071	0.81714
C	-4.79191	-0.45993	-0.79769
C	-5.66006	-2.54879	0.80311
H	-3.63140	-2.67620	1.48013
C	-6.12701	-0.83503	-0.80962
H	-4.45567	0.34548	-1.44149
C	-6.56576	-1.88232	-0.01044
H	-5.99545	-3.35970	1.44010
H	-6.82654	-0.31108	-1.45143
H	-7.60964	-2.17487	-0.01904
H	3.09317	1.00513	-0.13292

52			
**26**			
C	0.48884	3.11874	-0.15642
C	2.84132	2.85572	0.29590
C	3.00736	4.20018	0.23953
C	1.89861	5.04832	-0.02503
C	0.65888	4.51825	-0.21940
C	-0.19261	1.06853	-0.13778
C	1.16402	1.00321	0.12658

H	3.64452	2.16001	0.49062
H	3.99410	4.61555	0.39608
H	2.04645	6.12036	-0.07133
H	-0.20982	5.13100	-0.42178
N	1.59542	2.31945	0.09778
N	-0.58827	2.37257	-0.29777
C	-1.18534	-0.01248	-0.22199
C	-0.81491	-1.32771	-0.52217
C	-2.54923	0.25487	-0.00009
C	-1.74451	-2.34677	-0.54900
H	0.21614	-1.54687	-0.76613
C	-3.50345	-0.77580	-0.00624
C	-3.08064	-2.06623	-0.27699
H	-1.43844	-3.35835	-0.78969
H	-3.80199	-2.87438	-0.29599
O	-3.01716	1.49661	0.23951
C	2.10079	-0.10812	0.45717
H	2.76800	0.20500	1.26803
H	1.53043	-0.94288	0.86569
C	2.95452	-0.59738	-0.73076
H	2.29141	-0.95894	-1.52095
H	3.50075	0.24558	-1.16321
C	3.92417	-1.67923	-0.33005
C	5.25082	-1.37886	-0.03241
C	3.50360	-3.00277	-0.21256
C	6.13509	-2.36994	0.37340
H	5.60245	-0.35569	-0.12935
C	4.38219	-3.99737	0.19236
H	2.47519	-3.26090	-0.44755
C	5.70233	-3.68333	0.48830
H	7.16574	-2.11552	0.59549
H	4.03611	-5.02198	0.27327
H	6.39092	-4.45979	0.80217
C	-4.93924	-0.40172	0.26759
H	-4.98260	0.13110	1.22333
H	-5.25556	0.34044	-0.47184
C	-5.93773	-1.55866	0.28359
H	-5.95071	-2.06883	-0.68419
H	-5.62335	-2.30078	1.02721
C	-7.32252	-1.09437	0.61993
H	-7.43393	-0.59145	1.57980
C	-8.38924	-1.24161	-0.15267
H	-9.36596	-0.87942	0.14839
H	-8.32882	-1.73148	-1.12030

H	-2.30254	2.14830	0.07133

26			
**27**			
C	2.78803	0.66884	-0.00005
C	4.10110	-1.35611	0.00003
C	5.23949	-0.62221	0.00004
C	5.17549	0.79861	-0.00000
C	3.97280	1.43711	-0.00005
C	0.77922	-0.12393	-0.00001
C	1.61071	-1.21982	-0.00005
H	4.07174	-2.43706	0.00006
H	6.19447	-1.13045	0.00007
H	6.09444	1.37189	-0.00001
H	3.88782	2.51577	-0.00009
H	1.41729	-2.27673	-0.00005
N	2.88990	-0.71428	-0.00001
N	1.51775	1.03123	-0.00010
C	-0.68360	-0.05024	0.00002
C	-1.47232	-1.19847	0.00001
C	-1.34268	1.19771	0.00003
C	-2.84802	-1.16163	0.00001
C	-2.73741	1.22883	0.00001
C	-3.48310	0.06987	0.00000
O	-0.71402	2.37342	0.00006
F	-0.88674	-2.40757	0.00001
F	-3.56326	-2.28888	-0.00000
F	-4.81119	0.12847	-0.00000
F	-3.36843	2.40368	0.00002
H	0.26139	2.21783	0.00012

26			
**28**			
C	3.93073	-1.34337	-0.34714
C	4.48073	-0.10190	-0.02275
C	3.67283	0.98375	0.25096
C	2.28445	0.85497	0.20758
C	1.71271	-0.39298	-0.11700
C	2.55907	-1.47508	-0.39130
H	4.57198	-2.18968	-0.56113
H	5.55824	0.01584	0.01543
H	4.08885	1.95179	0.50286
H	2.11359	-2.43297	-0.64239
C	0.27430	-0.57801	-0.17397

H	-0.07899	-1.58143	-0.43744
C	-1.94198	0.12108	0.02943
C	-2.77213	1.07169	-0.56234
C	-2.50752	-1.01187	0.61343
C	-4.14209	0.87061	-0.60615
H	-2.32563	1.95994	-0.99422
C	-3.88185	-1.20224	0.57598
H	-1.87397	-1.72668	1.12666
C	-4.70309	-0.26754	-0.03861
H	-4.77747	1.61064	-1.07988
H	-4.31282	-2.08201	1.04107
H	-5.77635	-0.41760	-0.06404
O	1.53894	1.93193	0.47702
H	0.58698	1.68658	0.40304
N	-0.55469	0.36793	0.05218

25<br>**29**			
C	1.94585	-0.74551	-0.00009
C	1.90315	0.64324	-0.00011
C	3.08343	1.37557	0.00005
C	4.27245	0.66231	0.00025
C	4.28907	-0.73747	0.00027
C	3.11400	-1.47792	0.00009
C	-0.10941	-0.07531	-0.00022
H	3.06861	2.45853	0.00000
H	5.21393	1.19914	0.00037
H	5.24061	-1.25601	0.00041
H	3.11338	-2.56054	0.00008
C	-1.55090	-0.20402	-0.00004
C	-2.34951	0.95598	0.00003
C	-2.16377	-1.46330	0.00001
C	-3.73843	0.82461	0.00014
C	-3.53684	-1.58102	0.00012
H	-1.53749	-2.34698	-0.00006
C	-4.32094	-0.42605	0.00019
H	-4.33425	1.72921	0.00020
H	-4.00076	-2.55975	0.00014
H	-5.40230	-0.50827	0.00028
N	0.56730	1.03099	-0.00042
O	0.65367	-1.20068	-0.00038
O	-1.84122	2.19571	0.00004
H	-0.86128	2.16091	0.00016

25			
**30**			
C	2.14938	-0.73899	-0.00002
C	1.83317	0.62595	0.00004
C	2.85505	1.57497	0.00007
C	4.16634	1.14116	0.00005
C	4.47183	-0.22433	-0.00001
C	3.46895	-1.17734	-0.00005
C	-0.24633	-0.17420	0.00000
H	2.60732	2.62969	0.00011
H	4.97092	1.86734	0.00007
H	5.50821	-0.54161	-0.00002
H	3.70829	-2.23414	-0.00009
C	-1.70396	-0.15875	0.00001
C	-2.39795	1.06992	-0.00007
C	-2.44575	-1.34789	0.00006
C	-3.79430	1.06999	-0.00003
C	-3.82324	-1.33926	0.00006
H	-1.92395	-2.29893	0.00009
C	-4.49599	-0.11643	0.00003
H	-4.29847	2.02878	-0.00006
H	-4.37475	-2.27143	0.00010
H	-5.58021	-0.09534	0.00004
N	0.47912	0.90134	0.00002
O	-1.78304	2.25873	-0.00007
H	-0.80942	2.12400	-0.00003
S	0.67996	-1.67602	-0.00005

24			
**31**			
C	0.78833	-0.18988	-0.00000
C	-0.65658	-0.11586	-0.00000
C	-1.48579	-1.27055	-0.00000
C	1.55845	0.99467	0.00000
C	-1.27223	1.15976	-0.00000
C	-0.44879	2.33754	-0.00000
C	0.89713	2.26572	-0.00000
C	-2.66912	1.26991	-0.00000
C	-3.44764	0.13296	0.00000
C	-2.86301	-1.12955	0.00000
H	-3.46496	-2.03020	0.00000
H	-4.52893	0.21695	0.00001
H	-0.00257	-2.45777	0.00001
C	2.69645	-1.49243	-0.00000

C	3.53369	-0.37613	0.00000
C	2.95333	0.87049	0.00001
H	3.11135	-2.49569	-0.00001
H	4.60930	-0.50152	0.00000
H	-3.12509	2.25349	-0.00000
H	-0.94441	3.30259	-0.00000
H	1.50203	3.16641	0.00000
H	3.56606	1.76640	0.00001
N	1.38077	-1.40547	-0.00000
O	-0.98669	-2.51528	0.00000

29<br>**32**			
C	4.45146	-1.46711	-0.08592
C	3.36382	-1.75781	-0.89779
C	2.22699	-0.96294	-0.84938
C	2.17957	0.14102	-0.00034
C	3.28712	0.44488	0.78956
C	4.41086	-0.36485	0.75947
C	1.01624	1.07918	0.02926
C	-0.36133	0.58204	0.03645
C	-0.68204	-0.75389	0.30820
C	-1.98042	-1.21604	0.31747
H	-2.17797	-2.25385	0.54484
C	-3.01857	-0.31659	0.03751
C	-2.74267	1.01878	-0.21239
C	-1.43638	1.48234	-0.19279
H	5.33537	-2.09442	-0.11808
H	3.40063	-2.60406	-1.57455
H	3.25579	1.32352	1.42257
H	5.26222	-0.13046	1.38848
H	-3.55101	1.71248	-0.40390
H	1.38700	-1.18570	-1.49666
H	0.11479	-1.44772	0.54190
O	1.25282	2.28831	0.04599
O	-1.24310	2.78676	-0.40453
H	-0.29087	2.98274	-0.27233
O	-4.32269	-0.66192	0.00530
C	-4.68589	-2.00463	0.26310
H	-5.76893	-2.04088	0.17745
H	-4.24263	-2.68396	-0.46958
H	-4.39325	-2.30918	1.27127

36			

**33**			
C	-4.50598	-2.34689	-0.00995
C	-3.13422	-2.22166	-0.00083
C	-2.57340	-0.94431	-0.00035
C	-3.37689	0.19098	-0.00882
C	-4.76975	0.04110	-0.01796
C	-5.33157	-1.21434	-0.01856
H	-4.94962	-3.33615	-0.01037
H	-2.48128	-3.08584	0.00598
C	-2.73423	1.49612	-0.00768
H	-5.37684	0.93833	-0.02440
H	-6.40873	-1.33114	-0.02562
C	-1.28437	1.48439	0.00237
C	-0.56572	0.33151	0.01038
O	-1.22341	-0.86616	0.00873
O	-3.32594	2.57521	-0.01424
O	-0.71603	2.71227	0.00347
H	-1.46377	3.33834	-0.00243
C	0.88530	0.16753	0.02209
C	1.45035	-1.11316	0.03159
C	1.76757	1.25499	0.01879
C	2.81570	-1.30603	0.04213
H	0.80436	-1.98078	0.02744
C	3.13569	1.07126	0.02925
H	1.37775	2.26142	0.00469
C	3.70478	-0.21471	0.05059
H	3.19138	-2.31985	0.04338
H	3.76700	1.94887	0.02002
N	5.06788	-0.39852	0.08148
C	5.61539	-1.72999	-0.06466
H	5.34896	-2.18998	-1.02449
H	6.70028	-1.68060	-0.00416
H	5.27374	-2.39118	0.73575
C	5.94788	0.74004	-0.07381
H	5.79135	1.47472	0.71988
H	6.98113	0.40640	-0.00905
H	5.81295	1.24554	-1.03810

31			
**34**			
C	-4.59952	-1.00464	0.00040
C	-3.93961	0.21229	0.00060
C	-2.54896	0.24800	0.00030
C	-1.81844	-0.93969	-0.00017

C	-2.48828	-2.16058	-0.00039
C	-3.87241	-2.19165	-0.00009
C	-1.85208	1.55737	0.00058
C	-0.32943	-0.94113	-0.00041
C	0.38516	0.35342	-0.00034
C	-0.35842	1.55228	-0.00017
C	0.27083	2.78288	-0.00038
H	-0.33649	3.67833	-0.00011
C	1.65680	2.84076	-0.00082
C	2.41705	1.68929	-0.00098
C	1.80170	0.42955	-0.00076
H	-5.68324	-1.03252	0.00064
H	-4.48182	1.14966	0.00101
H	-1.90635	-3.07322	-0.00076
H	-4.39013	-3.14413	-0.00025
H	2.15794	3.80219	-0.00110
H	3.49406	1.73898	-0.00125
O	-2.47475	2.59775	0.00124
O	0.25464	-2.01900	-0.00129
N	2.55104	-0.74253	-0.00100
C	3.92123	-0.89944	0.00047
H	1.98618	-1.58597	-0.00131
O	4.71911	0.01251	0.00150
C	4.34620	-2.35023	0.00115
H	3.96595	-2.86804	0.88442
H	3.96171	-2.87065	-0.87872
H	5.43196	-2.39788	-0.00123

19			
**35**			
C	2.48293	0.87732	0.00025
C	1.09157	0.96743	0.00013
C	0.35122	-0.22320	-0.00018
C	0.99551	-1.45699	-0.00036
C	2.37472	-1.52423	-0.00022
C	3.11882	-0.34772	0.00009
H	3.04008	1.80639	0.00049
H	0.39202	-2.35440	-0.00060
H	2.86661	-2.48919	-0.00039
H	4.20215	-0.38767	0.00021
C	-3.04235	0.54195	-0.00018
H	-3.89343	-1.54010	0.00101
H	-3.84580	1.26023	-0.00035
N	-1.77490	0.93010	-0.00091

N	-1.07592	-0.19961	-0.00024
N	-1.81219	-1.29428	0.00071
C	-3.06290	-0.85330	0.00044
O	0.55512	2.20247	0.00039
H	-0.41867	2.14403	-0.00016

21			
**36**			
C	-3.69937	0.18794	-0.00004
C	-2.70226	1.14129	0.00012
C	-1.36961	0.72750	0.00009
C	-1.04607	-0.63478	-0.00007
C	-2.07635	-1.58380	-0.00024
C	-3.39169	-1.17778	-0.00022
H	-0.49000	2.61164	0.00032
H	-4.73594	0.50502	-0.00003
H	-2.94454	2.19880	0.00026
H	-1.79702	-2.63032	-0.00036
H	-4.18912	-1.91082	-0.00035
C	1.23037	-0.21476	0.00003
C	1.00749	1.25873	0.00015
N	0.26308	-1.05585	-0.00006
N	-0.31527	1.61486	0.00024
O	1.87885	2.11584	0.00014
C	2.63647	-0.81933	0.00006
O	2.78616	-2.01969	0.00082
N	3.65564	0.06731	-0.00088
H	4.59245	-0.30142	-0.00068
H	3.48596	1.06360	-0.00108

19			
**37**			
C	0.24049	3.99939	3.47528
C	-1.14680	4.04534	3.56392
C	-1.83683	2.95496	4.05927
C	-1.09760	1.83758	4.45484
C	0.28849	1.78601	4.36841
C	0.98181	2.88368	3.86982
C	0.77499	5.23658	2.93227
C	-0.39799	6.16192	2.64822
C	-1.66153	5.37768	3.06322
H	-2.91771	2.95561	4.14250
H	-1.62233	0.97225	4.84590
H	0.83912	0.90747	4.68184

H	-0.38944	6.43398	1.59077
H	-0.26445	7.08896	3.20953
O	1.95584	5.47375	2.74447
O	2.31909	2.86761	3.77275
H	2.62201	3.71850	3.40430
H	-2.22387	5.89503	3.84377
H	-2.34781	5.23839	2.22488

23<br>**38**			
C	-2.20245	-1.15112	-0.01705
C	-0.82028	-1.18347	-0.01680
C	-0.15785	-2.41798	-0.01699
C	-0.92985	-3.56551	-0.01742
C	-2.32989	-3.52730	-0.01768
C	-2.98532	-2.31806	-0.01749
H	-2.69896	-0.18680	-0.01690
H	-0.23522	-0.27182	-0.01646
H	-4.06674	-2.24975	-0.01768
C	-0.46179	-4.95737	-0.01769
C	-1.70121	-5.78947	-0.01817
C	-1.84140	-7.16080	-0.01859
C	-3.13131	-7.69045	-0.01898
C	-4.23546	-6.84746	-0.01894
C	-4.08814	-5.45886	-0.01852
C	-2.81186	-4.93236	-0.01814
H	-0.96746	-7.80256	-0.01861
H	-3.27518	-8.76475	-0.01931
H	-5.23197	-7.27531	-0.01925
H	-4.96289	-4.81801	-0.01851
O	0.70015	-5.32016	-0.01768
O	1.18370	-2.46511	-0.01673
H	1.47215	-3.39446	-0.01687

29<br>**39**			
C	-2.30347	-1.24282	0.10804
C	-0.91882	-1.23273	0.13105
C	-0.21936	-2.44089	0.05504
C	-0.95592	-3.61118	-0.04152
C	-2.35688	-3.61595	-0.06449
C	-3.04869	-2.42658	0.01048
H	-2.82918	-0.29600	0.16798
H	-0.36418	-0.30542	0.20700

H	-4.13152	-2.39072	-0.00440
C	-0.43416	-4.97828	-0.13255
C	-1.63695	-5.85345	-0.21533
C	-1.71964	-7.20427	-0.31475
C	-3.00242	-7.81667	-0.37975
C	-4.16624	-6.99890	-0.33899
C	-4.03693	-5.58245	-0.23408
C	-2.79917	-5.02536	-0.17384
H	-0.82177	-7.81313	-0.34459
H	-4.93567	-4.97482	-0.20430
O	0.74288	-5.29476	-0.13684
O	1.12237	-2.45202	0.07588
H	1.43653	-3.37185	0.01424
C	-4.39232	-9.79073	-0.54533
C	-5.54482	-8.98235	-0.50506
C	-5.43306	-7.62093	-0.40441
C	-3.15029	-9.21789	-0.48388
H	-4.49256	-10.86716	-0.62508
H	-6.52504	-9.44319	-0.55419
H	-6.32292	-7.00077	-0.37361
H	-2.25908	-9.83601	-0.51450

19<br>**40**			
C	-2.30219	-1.14923	0.11581
C	-0.92958	-1.27386	0.11972
C	-0.33737	-2.53617	0.04195
C	-1.15658	-3.67802	-0.04021
C	-2.54932	-3.52419	-0.04236
C	-3.12491	-2.27529	0.03459
H	-2.74551	-0.16121	0.17692
H	-0.27931	-0.40987	0.18227
H	-4.20276	-2.16835	0.03205
C	-0.53048	-5.00348	-0.12153
O	0.67561	-5.18891	-0.12300
O	1.00107	-2.59042	0.04996
H	1.28354	-3.52413	-0.00855
H	-3.16666	-4.41078	-0.10603
O	-1.39579	-6.01435	-0.19548
C	-0.83010	-7.32622	-0.27602
H	-1.67736	-8.00402	-0.32685
H	-0.21057	-7.41861	-1.16681
H	-0.22477	-7.53366	0.60511

25			
**41**			
C	-0.17801	-2.78111	-0.00003
C	-1.59384	-2.74960	-0.00002
C	-2.32708	-1.54729	0.00008
C	-1.56872	-0.38096	0.00016
C	-0.16983	-0.39260	0.00014
C	0.53372	-1.58053	0.00006
C	0.20695	-4.16205	-0.00017
C	-0.94334	-4.89063	-0.00016
H	-2.06072	0.58317	0.00024
H	0.36274	0.55168	0.00020
H	1.61850	-1.58086	0.00003
H	1.21158	-4.55632	-0.00023
H	-3.00942	-4.27315	-0.00005
C	-3.80727	-1.53479	0.00010
C	-4.54823	-0.34574	0.00021
C	-5.92761	-0.39792	0.00019
H	-4.05215	0.61448	0.00030
C	-5.76161	-2.76119	-0.00005
C	-6.56139	-1.63338	0.00007
H	-6.50563	0.51971	0.00028
H	-6.20687	-3.75213	-0.00015
H	-7.64083	-1.72103	0.00006
N	-2.02648	-4.04732	-0.00007
N	-4.43170	-2.72268	-0.00003
H	-1.08458	-5.96075	-0.00014

17			
**42**			
C	-1.21908	-0.50381	-0.01820
C	0.77598	0.65084	-0.24124
C	-1.31252	1.87523	-0.02147
C	-1.98174	0.69622	0.05788
H	-3.05645	0.67196	0.17466
C	-1.92218	-1.82361	0.06534
H	-1.20138	-2.63433	-0.00769
H	-2.46672	-1.91043	1.00922
H	-2.65521	-1.92060	-0.73982
C	-1.93950	3.22915	0.03880
H	-1.73443	3.79293	-0.87442
H	-3.01853	3.15083	0.15672
H	-1.54349	3.80333	0.87983
N	0.03399	1.82378	-0.16737

N	0.07959	-0.51960	-0.15909
S	2.42166	0.74732	-0.42034
H	0.57518	2.67559	-0.23093

28			
**43**			
C	-4.62337	-2.88105	0.00351
C	-3.25456	-2.85752	0.00281
C	-2.55393	-1.63934	0.00428
C	-3.24992	-0.40095	0.00654
C	-4.67036	-0.45858	0.00721
C	-5.32079	-1.67380	0.00571
C	-1.11965	-1.67715	0.00345
C	-2.43967	0.81960	0.00794
C	-1.02920	0.72547	0.00705
C	-0.38511	-0.55072	0.00476
C	-0.28203	1.91476	0.00847
H	0.80152	1.85599	0.00778
C	-0.91975	3.12719	0.01068
C	-2.31785	3.12645	0.01147
H	-0.63805	-2.64882	0.00171
H	-5.16372	-3.82016	0.00238
H	-2.68865	-3.78271	0.00111
H	-6.40557	-1.68920	0.00627
H	0.69886	-0.58909	0.00412
H	-0.37145	4.06105	0.01179
H	-2.86852	4.06275	0.01318
N	-3.03954	2.02772	0.01015
C	-5.57447	0.78224	0.00950
H	-4.93142	1.66863	0.01088
C	-6.40364	0.83887	1.21702
N	-7.02592	0.89231	2.17830
C	-6.40400	0.84301	-1.19757
N	-7.02656	0.89967	-2.15848

Cartesian coordinates optimized at the TD-CAM-B3LYP/6-311+G(d) level

26			
**1**			
C	1.95124	0.27266	-0.63568
C	3.20149	-0.77538	1.15188
C	4.35162	-0.32444	0.55158
C	4.34005	0.41760	-0.62521
C	3.08705	0.71910	-1.24493
C	-0.16034	-0.20808	0.03017
C	0.68287	-0.73678	0.93224
H	3.14824	-1.34407	2.06527
H	5.29431	-0.56398	1.02929
H	5.26274	0.76115	-1.06951
H	3.02435	1.27803	-2.16803
H	0.47539	-1.30183	1.82449
N	1.97732	-0.45539	0.53683
N	0.61858	0.38339	-0.97126
C	-1.63592	-0.23355	-0.04584
C	-2.30628	-1.29473	-0.59352
C	-2.38818	0.91259	0.44954
C	-3.70888	-1.32041	-0.65015
H	-1.74430	-2.13744	-0.97712
C	-3.83156	0.83995	0.38272
C	-4.46190	-0.24840	-0.15478
H	-4.20983	-2.18165	-1.07378
H	-4.37934	1.68797	0.77452
H	-5.54408	-0.28699	-0.20111
O	-1.81308	1.92128	0.89183
H	0.27833	1.10165	-1.58585

26			
**2**			
C	-3.45332	0.26419	0.64280
C	-4.70337	-0.85955	-1.09873
C	-5.85514	-0.42926	-0.48784
C	-5.84478	0.33903	0.67252
C	-4.59068	0.68959	1.26169
C	-1.34113	-0.16678	-0.05343
C	-2.18478	-0.74142	-0.92736
H	-4.65016	-1.44674	-2.00041
H	-6.79845	-0.70667	-0.94318
H	-6.76888	0.66576	1.12619
H	-4.52850	1.27003	2.17155
H	-1.97620	-1.32129	-1.80997

N	-3.47838	-0.49148	-0.51398
N	-2.11739	0.42193	0.95342
C	0.13426	-0.15001	-0.00743
C	0.85000	-1.19159	0.52218
C	0.84433	1.01673	-0.51523
C	2.25115	-1.18400	0.54744
H	0.32545	-2.05193	0.91870
C	2.29246	0.99035	-0.48497
C	2.95190	-0.08379	0.03466
H	2.79239	-2.02706	0.95429
O	0.23539	2.01069	-0.94034
H	2.81014	1.85161	-0.88535
Br	4.85051	-0.11358	0.07863
H	-1.79158	1.18179	1.52490

40			
**3**			
C	-3.62106	0.68448	-0.11120
C	-4.87089	-1.38534	-0.21445
C	-6.02342	-0.63991	-0.16802
C	-6.01348	0.74943	-0.09320
C	-4.75897	1.43512	-0.07265
C	-1.50821	-0.13455	-0.10416
C	-2.35163	-1.17859	-0.16634
H	-4.81712	-2.46022	-0.26274
H	-6.96686	-1.17258	-0.18903
H	-6.93817	1.30629	-0.05334
H	-4.69654	2.51354	-0.03197
H	-2.14316	-2.23344	-0.21470
N	-3.64604	-0.69467	-0.17645
N	-2.28590	1.03000	-0.11501
C	-0.03243	-0.08304	-0.09229
C	0.69947	-0.12958	-1.25019
C	0.66263	0.04884	1.18068
C	2.10033	-0.09713	-1.22975
H	0.18607	-0.20905	-2.20081
C	2.10851	0.06839	1.15406
C	2.81150	-0.00147	-0.01742
H	2.64315	-0.15493	-2.16516
H	2.62650	0.14447	2.10135
O	0.03637	0.16283	2.24869
N	4.24546	0.02380	0.00890
C	4.81901	1.20100	-0.64572
H	5.90432	1.09900	-0.57643

H	4.58079	1.22596	-1.72309
C	4.39764	2.50687	0.00574
H	4.65257	2.50794	1.06698
H	4.90885	3.34592	-0.47069
H	3.32418	2.68254	-0.08242
C	4.85624	-1.22283	-0.45686
H	4.61924	-1.42179	-1.51628
H	5.93778	-1.07762	-0.40870
C	4.47534	-2.42433	0.39079
H	4.73025	-2.25356	1.43820
H	3.40794	-2.64456	0.33606
H	5.01251	-3.31081	0.04738
H	-1.96133	1.91229	0.23974

27<br>**4**			
C	2.35061	0.23349	-0.65444
C	3.60201	-0.73614	1.17600
C	4.75281	-0.33143	0.54484
C	4.74111	0.34873	-0.66873
C	3.48694	0.63360	-1.29353
C	0.23829	-0.18325	0.05236
C	1.08311	-0.67571	0.97393
H	3.54933	-1.25638	2.11789
H	5.69618	-0.55804	1.02753
H	5.66428	0.65688	-1.13729
H	3.42376	1.14476	-2.24394
H	0.87576	-1.19116	1.89580
N	2.37692	-0.43343	0.55448
N	1.01660	0.34320	-0.98625
C	-1.23609	-0.19750	-0.01180
C	-1.92528	-1.27581	-0.50882
C	-1.98080	0.97475	0.43920
C	-3.32024	-1.29502	-0.55534
H	-1.37727	-2.14076	-0.86083
C	-3.42302	0.91688	0.38381
C	-4.06278	-0.18777	-0.09987
H	-3.85632	-2.15575	-0.93258
O	-1.39338	1.99239	0.83772
H	-3.96057	1.78940	0.73861
O	-5.41190	-0.30825	-0.18358
H	-5.84870	0.48309	0.14887
H	0.68111	1.03755	-1.63032

30			
**5**			
C	-2.75449	0.21582	0.66180
C	-4.01572	-0.59977	-1.23567
C	-5.16056	-0.20350	-0.58845
C	-5.14102	0.39764	0.66603
C	-3.88507	0.60637	1.31682
C	-0.64815	-0.21569	-0.05429
C	-1.49806	-0.62395	-1.01173
H	-3.96900	-1.05998	-2.20862
H	-6.10541	-0.37216	-1.09156
H	-6.05947	0.70154	1.14653
H	-3.81627	1.05387	2.29845
H	-1.29677	-1.08532	-1.96312
N	-2.78836	-0.37180	-0.58742
N	-1.42109	0.26585	1.00992
C	0.82428	-0.27547	0.02050
C	1.47898	-1.40102	0.46101
C	1.60676	0.89759	-0.35418
C	2.86932	-1.45906	0.51712
H	0.90428	-2.26936	0.75807
C	3.04901	0.80121	-0.29280
C	3.65482	-0.34769	0.13157
H	3.37918	-2.35401	0.84866
H	3.59631	1.68294	-0.59428
O	1.05070	1.95273	-0.69638
O	4.98872	-0.54151	0.22937
C	5.84502	0.52277	-0.14086
H	5.67608	1.40073	0.48801
H	6.85780	0.15862	0.00864
H	5.70522	0.79317	-1.19066
H	-1.07195	0.91018	1.69730

26			
**6**			
C	-2.24682	0.02159	-0.69362
C	-3.47963	-0.47451	1.32916
C	-4.63701	-0.35271	0.60021
C	-4.63779	-0.04839	-0.75750
C	-3.38902	0.13697	-1.42867
C	-0.13292	-0.01922	0.11833
C	-0.96625	-0.28841	1.13674
H	-3.41801	-0.69871	2.38106

H	-5.57580	-0.50065	1.12075
H	-5.56628	0.04457	-1.30145
H	-3.33452	0.35756	-2.48551
H	-0.74994	-0.50303	2.16909
N	-2.26129	-0.27234	0.65590
N	-0.91376	0.12998	-1.03459
C	1.34156	0.06191	0.07224
C	2.10618	-1.05998	-0.10066
C	1.98226	1.36791	0.18203
C	3.49754	-0.95093	-0.12981
H	1.65519	-2.03866	-0.20146
C	3.42973	1.40101	0.15583
C	4.16967	0.26342	0.00133
H	3.89657	2.37193	0.26119
H	5.25232	0.27549	-0.02299
O	1.31460	2.41042	0.27313
F	4.21509	-2.06612	-0.28082
H	-0.61194	0.64779	-1.84126

26			
**7**			
C	-2.54775	-0.03827	-0.69347
C	-3.74182	-0.62204	1.32936
C	-4.90112	-0.61601	0.59357
C	-4.92177	-0.32911	-0.76792
C	-3.69151	-0.03775	-1.43523
C	-0.44370	0.12518	0.12922
C	-1.25507	-0.20820	1.14670
H	-3.66608	-0.82717	2.38423
H	-5.82538	-0.84352	1.11135
H	-5.85167	-0.32809	-1.31734
H	-3.65129	0.17434	-2.49443
H	-1.02577	-0.39119	2.18236
N	-2.54326	-0.31660	0.65965
N	-1.22843	0.18872	-1.02915
C	1.01742	0.33824	0.09061
C	1.88112	-0.71491	-0.03143
C	1.53519	1.70066	0.15390
C	3.26851	-0.50063	-0.05090
H	1.50220	-1.72627	-0.09886
C	2.97414	1.85985	0.14276
C	3.81232	0.78782	0.04171
H	3.35407	2.87115	0.21509
H	4.88767	0.91418	0.03058

O	0.77625	2.68033	0.19312
Cl	4.32489	-1.85789	-0.17795
H	-0.97066	0.72420	-1.83958

26			
**8**			
C	3.14120	-0.19242	0.67771
C	4.42276	0.00661	-1.36599
C	5.50103	-0.50514	-0.68717
C	5.43696	-0.86722	0.65497
C	4.20787	-0.69324	1.36385
C	1.12807	0.54829	-0.05031
C	1.98120	0.59893	-1.08698
H	4.41017	0.29378	-2.40424
H	6.42776	-0.62668	-1.23538
H	6.30242	-1.27187	1.15882
H	4.11094	-0.94240	2.41121
H	1.81973	0.92571	-2.09978
N	3.21672	0.15388	-0.65728
N	1.84891	0.09881	1.06287
C	-0.29646	0.92707	0.04489
C	-0.68162	2.22356	0.22733
C	-1.29715	-0.13562	-0.03155
C	-2.04265	2.57582	0.29494
H	0.07189	2.99729	0.30483
C	-2.69266	0.28842	0.02923
C	-3.03819	1.60792	0.19088
H	-2.32188	3.61416	0.42053
H	-4.08151	1.89308	0.23763
O	-0.96782	-1.32187	-0.11852
Br	-4.01419	-1.02960	-0.11754
H	1.41816	-0.33880	1.85837

27			
**9**			
C	2.44854	0.09747	-0.66227
C	3.59527	-0.90413	1.22007
C	4.77215	-0.73729	0.53542
C	4.82511	-0.16378	-0.73290
C	3.61128	0.25844	-1.35467
C	0.32402	0.07207	0.11780
C	1.11044	-0.47187	1.06174
H	3.49438	-1.33222	2.20364
H	5.68470	-1.06779	1.01699

H	5.76891	-0.04082	-1.24333
H	3.59679	0.69160	-2.34501
H	0.85432	-0.88025	2.02437
N	2.41188	-0.46692	0.59950
N	1.13579	0.38940	-0.97884
C	-1.13839	0.27294	0.08832
C	-1.99859	-0.76341	-0.12057
C	-1.66491	1.62565	0.26592
C	-3.39978	-0.56223	-0.10403
H	-1.61455	-1.76251	-0.28235
C	-3.10536	1.78587	0.28804
C	-3.93881	0.72318	0.10766
H	-3.48322	2.78704	0.45215
H	-5.01439	0.84956	0.12261
O	-0.90607	2.59745	0.36578
C	-4.26822	-1.67008	-0.29071
N	-4.97280	-2.56724	-0.44307
H	0.90112	1.11208	-1.63739

29			
**10**			
C	-2.25821	0.00796	0.69268
C	-3.48731	-0.50211	-1.32867
C	-4.64519	-0.40404	-0.59617
C	-4.64793	-0.10545	0.76258
C	-3.40091	0.09988	1.43224
C	-0.14442	0.00487	-0.12347
C	-0.97566	-0.27393	-1.14089
H	-3.42388	-0.71993	-2.38177
H	-5.58267	-0.56622	-1.11493
H	-5.57687	-0.03086	1.30875
H	-3.34749	0.31837	2.48951
H	-0.75849	-0.47892	-2.17491
N	-2.27116	-0.28072	-0.65681
N	-0.92777	0.13904	1.02870
C	1.32915	0.11203	-0.07772
C	2.11718	-0.99228	0.08225
C	1.94312	1.43012	-0.17269
C	3.52697	-0.91590	0.11799
H	1.64720	-1.96579	0.17024
C	3.38678	1.47919	-0.14515
C	4.13576	0.34391	-0.00265
H	3.84291	2.45707	-0.23823
H	5.21843	0.40952	0.01927

O	1.25762	2.46335	-0.25030
C	4.33816	-2.16341	0.26299
H	4.20688	-2.81835	-0.60360
H	5.40179	-1.94734	0.35944
H	4.02686	-2.73780	1.13935
H	-0.62784	0.64029	1.84611

30			
**11**			
C	-2.60598	-0.11608	-0.69787
C	-3.82986	-0.52112	1.35014
C	-4.97977	-0.57395	0.59994
C	-4.98076	-0.40450	-0.78053
C	-3.74001	-0.17493	-1.45488
C	-0.51028	0.10168	0.13534
C	-1.33692	-0.13664	1.16636
H	-3.76857	-0.63428	2.41962
H	-5.91218	-0.75291	1.12232
H	-5.90361	-0.44714	-1.34034
H	-3.68429	-0.05340	-2.52744
H	-1.12324	-0.22910	2.21707
N	-2.62131	-0.27725	0.67209
N	-1.28321	0.07629	-1.03062
C	0.95389	0.30465	0.09172
C	1.80495	-0.75456	0.00587
C	1.47784	1.66568	0.10459
C	3.20569	-0.57233	-0.03306
H	1.42997	-1.76998	-0.01964
C	2.91542	1.80223	0.07828
C	3.75155	0.72108	0.00973
H	3.30699	2.81130	0.11207
H	4.82299	0.86989	-0.01165
O	0.72874	2.65623	0.11606
O	3.92667	-1.69952	-0.10024
C	5.34303	-1.62098	-0.12522
H	5.69524	-2.64706	-0.17595
H	5.68897	-1.07256	-1.00379
H	5.72447	-1.14845	0.78227
H	-1.00118	0.50538	-1.89395

26			
**12**			
C	-1.11364	0.79411	-0.59252
C	-2.54715	-0.87404	0.41994

C	-3.61482	-0.09827	0.03873
C	-3.49020	1.10247	-0.64127
C	-2.17747	1.55893	-0.97618
C	0.91076	-0.10947	-0.13454
C	-0.02526	-0.93093	0.36975
H	-2.60952	-1.80441	0.95651
H	-4.36441	1.67344	-0.91270
H	-2.02229	2.47940	-1.51998
H	0.08197	-1.86117	0.89993
N	-1.26864	-0.39137	0.09343
N	0.24204	0.94355	-0.76395
C	2.38529	-0.20897	-0.13948
C	3.05154	-0.92097	-1.10066
C	3.14089	0.49273	0.89036
C	4.45206	-1.02119	-1.09212
H	2.48816	-1.42899	-1.87379
C	4.58062	0.35347	0.86864
C	5.20626	-0.38262	-0.09973
H	4.94943	-1.60495	-1.85621
H	5.12993	0.86163	1.65134
H	6.28608	-0.47527	-0.10490
O	2.57284	1.19866	1.74028
Cl	-5.20932	-0.68514	0.45091
H	0.66919	1.82148	-0.99979

26			
**13**			
C	0.37806	1.02102	-0.47536
C	1.89884	-0.75261	0.16271
C	2.92612	0.12780	-0.07473
C	2.73987	1.43314	-0.50085
C	1.40234	1.89062	-0.71776
C	-1.59896	-0.03898	-0.17169
C	-0.62045	-0.90369	0.14503
H	2.00466	-1.76822	0.50125
H	3.58138	2.08685	-0.66874
H	1.19901	2.89286	-1.06622
H	-0.67949	-1.92362	0.48304
N	0.59438	-0.26876	-0.03981
N	-0.98563	1.14438	-0.59103
C	-3.06828	-0.19659	-0.17133
C	-3.73120	-0.73402	-1.24212
C	-3.82362	0.25860	0.98892
C	-5.12631	-0.89184	-1.22937

H	-3.16870	-1.05749	-2.10939
C	-5.25725	0.06683	0.96491
C	-5.87886	-0.49119	-0.11821
H	-5.62047	-1.33520	-2.08448
H	-5.80549	0.38907	1.84137
H	-6.95425	-0.62570	-0.12301
O	-3.26114	0.80785	1.95084
Br	4.69374	-0.51479	0.20915
H	-1.45157	2.03249	-0.64597

27			
**14**			
C	1.25455	0.81847	-0.51154
C	2.67872	-0.92730	0.36774
C	3.76572	-0.12575	0.04359
C	3.62479	1.12480	-0.54439
C	2.32130	1.61632	-0.84008
C	-0.77288	-0.11333	-0.14297
C	0.15953	-0.97415	0.30029
H	2.73157	-1.89373	0.83676
H	4.49920	1.71372	-0.77766
H	2.16509	2.57748	-1.30643
H	0.04973	-1.94050	0.76044
N	1.40407	-0.41323	0.07896
N	-0.09096	0.98726	-0.66573
C	-2.24815	-0.20045	-0.15805
C	-2.91872	-0.80545	-1.18658
C	-2.99731	0.39472	0.94109
C	-4.32071	-0.89317	-1.18842
H	-2.35988	-1.23777	-2.00764
C	-4.43832	0.27254	0.90412
C	-5.06965	-0.35339	-0.13575
H	-4.82236	-1.39050	-2.00869
H	-4.98386	0.70139	1.73537
H	-6.15020	-0.43495	-0.15052
O	-2.42304	0.99766	1.86264
C	5.07669	-0.62513	0.33362
N	6.12675	-1.02914	0.56570
H	-0.51724	1.85699	-0.93124

29			
**15**			
C	1.50308	0.57591	0.67107
C	2.87299	-0.87748	-0.69489

C	3.99207	-0.24421	-0.21585
C	3.88396	0.80303	0.70768
C	2.59837	1.21491	1.16892
C	-0.55556	-0.15930	0.07943
C	0.34775	-0.88433	-0.60034
H	2.87660	-1.68334	-1.41154
H	4.77338	1.29734	1.07374
H	2.48144	2.00768	1.89486
H	0.20206	-1.68694	-1.30272
N	1.61176	-0.45071	-0.24575
N	0.14923	0.72038	0.91110
C	-2.03149	-0.22633	0.09670
C	-2.69951	-1.13355	0.87558
C	-2.78875	0.71875	-0.71436
C	-4.10150	-1.20227	0.87415
H	-2.13482	-1.82144	1.49284
C	-4.23072	0.60548	-0.69599
C	-4.85735	-0.33003	0.08036
H	-4.59981	-1.94276	1.48696
H	-4.78077	1.29769	-1.32128
H	-5.93888	-0.40085	0.08686
O	-2.21886	1.60220	-1.37714
C	5.34556	-0.68776	-0.70044
H	5.87199	0.13463	-1.19000
H	5.96759	-1.02217	0.13293
H	5.27146	-1.50961	-1.41386
H	-0.23963	1.58749	1.23865

29<br>**16**			
C	0.44658	0.98124	0.51909
C	1.95094	-0.73774	-0.27734
C	2.99398	0.11218	0.02284
C	2.80435	1.37957	0.55421
C	1.47571	1.82968	0.81967
C	-1.53734	-0.04772	0.15993
C	-0.56625	-0.88658	-0.23741
H	2.04479	-1.71606	-0.71433
H	3.64927	2.01736	0.76453
H	1.27791	2.80301	1.24377
H	-0.63228	-1.87510	-0.65723
N	0.65277	-0.26945	-0.02093
N	-0.91096	1.09860	0.65599
C	-3.00768	-0.19826	0.16473

C	-3.66145	-0.80387	1.20378
C	-3.77212	0.33593	-0.95507
C	-5.05803	-0.95294	1.19634
H	-3.09185	-1.18860	2.04094
C	-5.20664	0.15130	-0.92727
C	-5.81992	-0.47450	0.12332
H	-5.54539	-1.45001	2.02538
H	-5.76246	0.53413	-1.77406
H	-6.89600	-0.60305	0.13122
O	-3.21633	0.94195	-1.88608
C	4.38774	-0.39885	-0.17655
F	5.24387	0.57361	-0.53373
F	4.45575	-1.35196	-1.12638
F	4.88815	-0.95151	0.94955
H	-1.37524	1.97063	0.83631

36			
**17**			
C	-0.15432	1.21991	0.27823
C	1.46818	-0.48625	-0.28018
C	2.47407	0.38315	0.08965
C	2.17933	1.66680	0.55522
C	0.82212	2.10179	0.63776
C	-2.06943	0.09381	-0.15989
C	-1.04032	-0.73057	-0.42113
H	1.62051	-1.46261	-0.70975
H	2.97497	2.31621	0.89070
H	0.56206	3.09406	0.97873
H	-1.04124	-1.74523	-0.78028
N	0.13643	-0.05409	-0.16215
N	-1.52734	1.32154	0.23589
C	-3.52701	-0.10989	-0.28367
C	-4.17517	0.05373	-1.47941
C	-4.29129	-0.46782	0.90493
C	-5.55789	-0.15830	-1.59290
H	-3.60767	0.33701	-2.35735
C	-5.71163	-0.68798	0.74084
C	-6.31601	-0.53327	-0.47613
H	-6.03849	-0.03828	-2.55555
H	-6.26432	-0.97575	1.62647
H	-7.38177	-0.69856	-0.58320
O	-3.74824	-0.55879	2.01875
C	3.88387	-0.07367	-0.01584
C	4.26687	-1.33444	0.44120

C	4.85755	0.75290	-0.57639
C	5.58451	-1.75822	0.33935
H	3.52594	-1.97807	0.90234
C	6.17560	0.33161	-0.67744
H	4.57046	1.72906	-0.95083
C	6.54447	-0.92664	-0.22075
H	5.86379	-2.73918	0.70839
H	6.91684	0.98707	-1.12176
H	7.57451	-1.25643	-0.29957
H	-2.03424	2.00913	0.76475

36			
**18**			
C	-0.78003	0.97920	0.18098
C	-0.90275	3.39637	0.10319
C	-2.26986	3.27416	0.02004
C	-2.90771	2.04390	0.01559
C	-2.15187	0.82489	0.11842
C	1.46506	0.71148	0.16744
C	1.20923	2.03110	0.15743
H	-0.34216	4.31586	0.09552
H	-2.85721	4.18301	-0.03542
H	-3.98695	1.99560	-0.01128
H	1.88367	2.86957	0.14461
N	-0.16291	2.20582	0.16841
N	0.23511	0.05781	0.21721
C	2.74602	-0.02383	0.17425
C	3.41008	-0.29545	1.34057
C	3.30056	-0.48263	-1.09390
C	4.63365	-0.98431	1.33836
H	2.99078	0.03724	2.28220
C	4.56702	-1.18090	-1.05035
C	5.20427	-1.41995	0.13607
H	5.14076	-1.17277	2.27601
H	4.97675	-1.50236	-1.99968
H	6.15134	-1.94651	0.15243
O	2.70410	-0.29505	-2.16673
C	-2.78211	-0.49735	0.08720
C	-3.92845	-0.71673	-0.69145
C	-2.29890	-1.57900	0.83957
C	-4.54454	-1.95593	-0.73099
H	-4.32273	0.09516	-1.29149
C	-2.91620	-2.82032	0.79592
H	-1.46498	-1.43186	1.51720

C	-4.04096	-3.02104	0.00729
H	-5.42321	-2.09515	-1.35185
H	-2.52513	-3.63260	1.39946
H	-4.52434	-3.99061	-0.02537
H	0.09021	-0.91157	-0.01157

40			
**19**			
C	1.02724	1.11052	0.72505
C	-0.66763	-0.45136	-0.01371
C	-1.63720	0.44465	0.38784
C	-1.28660	1.67198	0.95764
C	0.08865	2.01287	1.13006
C	2.89153	-0.04573	0.16445
C	1.82762	-0.79520	-0.17230
H	-0.85793	-1.37385	-0.53737
H	-2.05594	2.34261	1.31221
H	0.39072	2.94806	1.58048
H	1.78495	-1.77319	-0.61993
N	0.68121	-0.10333	0.16858
N	2.40516	1.12240	0.76303
C	4.33883	-0.31246	0.04151
C	5.01447	-1.05384	0.97467
C	5.06438	0.25326	-1.08904
C	6.38616	-1.31798	0.84010
H	4.47669	-1.45706	1.82395
C	6.47398	-0.05343	-1.19562
C	7.10550	-0.81597	-0.25226
H	6.88836	-1.92268	1.58458
H	6.99646	0.35274	-2.05276
H	8.16306	-1.03492	-0.34336
O	4.49903	0.98913	-1.91556
C	-3.06427	0.08164	0.19716
C	-3.54077	-1.19084	0.52603
C	-3.97354	0.99766	-0.31679
C	-4.86736	-1.53034	0.34633
H	-2.85862	-1.91867	0.95129
C	-5.31351	0.67280	-0.50312
H	-3.62802	1.98628	-0.59747
C	-5.76491	-0.59864	-0.17080
H	-5.23645	-2.51422	0.61112
H	-5.98364	1.41626	-0.91403
O	-7.05103	-1.02250	-0.31226
C	-8.00292	-0.11510	-0.82065

H	-7.74806	0.20666	-1.83498
H	-8.94911	-0.65097	-0.84290
H	-8.10212	0.76386	-0.17649
H	2.93310	1.97568	0.81771

42			
**20**			
C	1.75591	1.16719	-0.71513
C	0.00611	-0.35672	-0.02840
C	-0.92891	0.57881	-0.42881
C	-0.53542	1.80373	-0.96942
C	0.85114	2.10503	-1.12268
C	3.57735	-0.05394	-0.15347
C	2.48729	-0.77462	0.16274
H	-0.21910	-1.28068	0.47788
H	-1.27905	2.50446	-1.32065
H	1.18735	3.03615	-1.55626
H	2.40899	-1.75557	0.59862
N	1.36704	-0.04052	-0.17782
N	3.13001	1.13748	-0.73264
C	5.01505	-0.36257	-0.01534
C	5.68574	-1.09601	-0.95793
C	5.73552	0.15148	1.14278
C	7.04843	-1.39834	-0.81003
H	5.15179	-1.46283	-1.82595
C	7.13534	-0.19321	1.26152
C	7.76256	-0.94486	0.30649
H	7.54701	-1.99476	-1.56350
H	7.65416	0.17537	2.13762
H	8.81277	-1.19281	0.40732
O	5.17453	0.87483	1.98287
C	-2.36894	0.25555	-0.27305
C	-2.86320	-1.00604	-0.60960
C	-3.26243	1.20889	0.21748
C	-4.20579	-1.30577	-0.45999
H	-2.18718	-1.75006	-1.01486
C	-4.60717	0.91503	0.36682
H	-2.89048	2.18593	0.50284
C	-5.08854	-0.34742	0.02929
H	-4.59023	-2.28221	-0.72831
H	-5.28787	1.66176	0.75439
C	-6.51878	-0.71792	0.17069
O	-6.97783	-1.79721	-0.10773
O	-7.27109	0.28724	0.64965

C	-8.65975	0.00009	0.81224
H	-9.10446	0.91175	1.20219
H	-9.10931	-0.26609	-0.14383
H	-8.80026	-0.82336	1.51169
H	3.69239	1.96509	-0.82131

40			
**21**			
C	0.14030	1.43783	0.16819
C	0.70443	3.78817	0.00822
C	-0.64205	4.05540	-0.04812
C	-1.60332	3.05594	0.00116
C	-1.21465	1.67822	0.13217
C	2.21914	0.54394	0.13426
C	2.34687	1.88109	0.08182
H	1.49978	4.51305	-0.03921
H	-0.94905	5.09171	-0.12644
H	-2.65339	3.31159	-0.00530
H	3.23203	2.49144	0.03303
N	1.08318	2.43999	0.10648
N	0.85466	0.26401	0.22906
C	3.24053	-0.52229	0.15254
C	3.83864	-0.92282	1.31800
C	3.60142	-1.17270	-1.10158
C	4.81887	-1.92731	1.32549
H	3.55952	-0.44703	2.25011
C	4.62252	-2.19633	-1.04908
C	5.20550	-2.55546	0.13469
H	5.28305	-2.21220	2.26109
H	4.89458	-2.65927	-1.98939
H	5.96716	-3.32609	0.15839
O	3.04379	-0.87215	-2.16989
C	-2.19591	0.58391	0.16040
C	-3.34773	0.63911	-0.62502
C	-2.04591	-0.54022	0.98772
C	-4.30004	-0.37296	-0.60998
H	-3.49901	1.48754	-1.28262
C	-2.98128	-1.55629	1.00808
H	-1.20101	-0.59841	1.66535
C	-4.11733	-1.48353	0.20552
H	-5.17116	-0.28282	-1.24566
H	-2.86164	-2.41392	1.65982
O	-4.98352	-2.53165	0.29452
C	-6.15482	-2.49785	-0.48918

H	-6.69527	-3.41464	-0.26485
H	-6.78282	-1.63865	-0.23428
H	-5.92145	-2.47053	-1.55796
H	0.44058	-0.62449	0.00031

42			
**22**			
C	-1.03395	1.60538	-0.08417
C	-2.00003	3.81676	-0.00570
C	-0.72827	4.32244	0.05614
C	0.39437	3.49670	0.03592
C	0.29767	2.08692	-0.06646
C	-2.89769	0.36387	-0.00079
C	-3.27472	1.66140	-0.01201
H	-2.91914	4.37906	0.00448
H	-0.60110	5.39659	0.10862
H	1.37491	3.95254	0.05179
H	-4.24540	2.12334	0.02210
N	-2.11088	2.42852	-0.06535
N	-1.51913	0.34514	-0.05249
C	-3.69645	-0.87316	0.01899
C	-5.02483	-0.86107	-0.32400
C	-3.06341	-2.12746	0.40303
C	-5.80320	-2.02662	-0.27373
H	-5.49140	0.06331	-0.64555
C	-3.89860	-3.29914	0.43726
C	-5.22946	-3.24121	0.11186
H	-6.85117	-1.97969	-0.54187
H	-3.41842	-4.22418	0.73132
H	-5.83838	-4.13721	0.14502
O	-1.84435	-2.19066	0.68438
C	1.44004	1.20097	-0.09812
C	2.65657	1.58424	0.51089
C	1.42361	-0.04746	-0.76041
C	3.76484	0.76931	0.48300
H	2.71071	2.52325	1.04889
C	2.53683	-0.85854	-0.79072
H	0.55140	-0.35624	-1.32315
C	3.72527	-0.47151	-0.16597
H	4.67603	1.08123	0.97722
H	2.51085	-1.80508	-1.31670
C	4.88161	-1.37690	-0.22874
O	4.89379	-2.45208	-0.78198
O	5.96732	-0.88519	0.40734

C	7.12353	-1.71726	0.38621
H	7.44894	-1.89810	-0.63810
H	7.88757	-1.17444	0.93715
H	6.91738	-2.67477	0.86419
H	-0.99907	-0.50777	0.12683

29			
**23**			
C	1.43798	0.26099	0.96078
C	2.84444	-0.41132	-0.89191
C	3.92467	-0.17668	-0.07625
C	3.81981	0.26771	1.23179
C	2.51373	0.48583	1.77113
C	-0.59798	0.05855	-0.00840
C	0.32433	-0.28562	-0.92267
H	2.89226	-0.74699	-1.91288
H	4.70367	0.44062	1.82577
H	2.37217	0.81541	2.79014
H	0.20294	-0.60994	-1.94146
N	1.57458	-0.16985	-0.34114
N	0.08383	0.36432	1.17216
C	-2.07360	0.09854	-0.08738
C	-2.82858	-1.01392	0.15330
C	-2.72581	1.36390	-0.39772
C	-4.23874	-1.00005	0.07404
H	-2.33134	-1.94594	0.39928
C	-4.16776	1.34767	-0.48375
C	-4.88232	0.20478	-0.25240
H	-4.65169	2.28317	-0.73578
H	-5.96490	0.22139	-0.31978
O	-2.07398	2.40913	-0.55806
Cl	5.50970	-0.46247	-0.75693
C	-5.01170	-2.25614	0.31842
H	-4.77630	-3.01339	-0.43532
H	-4.76064	-2.69195	1.28899
H	-6.08707	-2.08277	0.29292
H	-0.31185	0.89258	1.92927

32			
**24**			
C	-0.77317	-0.43020	1.04173
C	-2.25325	0.30712	-0.72394
C	-3.30959	-0.00137	0.10678
C	-3.13883	-0.52166	1.38126

C	-1.81550	-0.73825	1.87175
C	1.22544	-0.10085	0.03100
C	0.26627	0.26746	-0.83428
H	-2.33406	0.68580	-1.72739
H	-3.99437	-0.75972	1.99467
H	-1.63136	-1.12981	2.86123
H	0.34610	0.65407	-1.83520
N	-0.96168	0.07288	-0.22663
N	0.58412	-0.50686	1.20411
C	2.69887	-0.09197	-0.08975
C	3.42919	1.02246	0.20965
C	3.37527	-1.31202	-0.50943
C	4.83680	1.05510	0.09320
H	2.91419	1.92024	0.53406
C	4.81351	-1.24812	-0.63026
C	5.50309	-0.10454	-0.33526
H	5.31528	-2.14878	-0.96176
H	6.58343	-0.08510	-0.43131
O	2.74709	-2.36008	-0.73203
C	-4.69118	0.31072	-0.37971
F	-5.60638	-0.55810	0.08287
F	-4.77920	0.29677	-1.72417
F	-5.09918	1.53727	0.01229
C	5.58194	2.31271	0.40574
H	5.33783	2.67884	1.40632
H	6.66067	2.17000	0.35070
H	5.31360	3.11017	-0.29363
H	1.02444	-1.02020	1.94647

46			
**25**			
C	-0.18937	0.17404	-0.20410
C	1.39559	-1.65564	-0.20931
C	2.41756	-0.73178	-0.09737
C	2.14362	0.63058	-0.03688
C	0.80173	1.13398	-0.11618
C	-2.12149	-0.99701	-0.21583
C	-1.10588	-1.87848	-0.24226
H	1.52050	-2.71911	-0.32525
H	2.96120	1.33086	0.05740
H	-1.11954	-2.95431	-0.26425
N	0.08155	-1.16938	-0.23731
N	-1.55551	0.27586	-0.22444
C	-3.58289	-1.20745	-0.21740

C	-4.28283	-1.37196	-1.38305
C	-4.29146	-1.22212	1.05703
C	-5.67042	-1.58491	-1.37431
H	-3.75567	-1.35006	-2.32895
C	-5.71917	-1.45323	1.01952
C	-6.37801	-1.62647	-0.16642
H	-6.19394	-1.72449	-2.31145
H	-6.23191	-1.47874	1.97283
H	-7.44827	-1.79618	-0.17830
O	-3.69666	-1.02937	2.12980
C	0.49048	2.56221	-0.02686
C	1.26857	3.40985	0.77663
C	-0.56369	3.14697	-0.74611
C	0.99313	4.76321	0.87098
H	2.08452	2.99041	1.35357
C	-0.83886	4.50275	-0.64764
H	-1.13780	2.54536	-1.44241
C	-0.06655	5.32177	0.16490
H	1.60723	5.38877	1.51007
H	-1.65278	4.92570	-1.22700
H	-0.28057	6.38159	0.24064
C	3.81979	-1.21950	-0.04911
C	4.17403	-2.30221	0.75586
C	4.81412	-0.60481	-0.81016
C	5.48428	-2.75720	0.79996
H	3.41679	-2.77563	1.37098
C	6.12487	-1.05752	-0.76584
H	4.55042	0.22564	-1.45531
C	6.46540	-2.13639	0.03901
H	5.74117	-3.59492	1.43923
H	6.88248	-0.56925	-1.36920
H	7.48969	-2.49074	0.07352
H	-2.03783	1.12263	0.02730

52			
**26**			
C	-1.32564	3.28490	0.20771
C	-3.30241	2.30126	-0.77155
C	-3.82559	3.56711	-0.88163
C	-3.14008	4.69985	-0.45699
C	-1.83832	4.54517	0.11141
C	-0.05554	1.43328	0.48543
C	-1.22921	1.03238	-0.04281
H	-3.78739	1.40071	-1.10574

H	-4.81092	3.66509	-1.32215
H	-3.57839	5.68201	-0.55757
H	-1.26177	5.39062	0.46039
N	-2.02029	2.16494	-0.20689
N	-0.12451	2.82009	0.69520
C	1.14165	0.66208	0.87510
C	1.24750	0.06583	2.10262
C	2.25125	0.57243	-0.06705
C	2.39317	-0.66490	2.44888
H	0.43366	0.15241	2.81210
C	3.42871	-0.20847	0.31478
C	3.46133	-0.79718	1.55474
H	2.44949	-1.13974	3.42064
H	4.32436	-1.37588	1.86059
O	2.20831	1.15990	-1.15929
C	-1.71191	-0.33322	-0.38934
H	-2.16297	-0.32305	-1.38641
H	-0.85157	-1.00031	-0.46063
C	-2.71907	-0.90947	0.62460
H	-2.23560	-0.96554	1.60355
H	-3.56078	-0.22249	0.73876
C	-3.22059	-2.27155	0.21898
C	-4.39018	-2.41131	-0.52423
C	-2.50562	-3.42146	0.54768
C	-4.83370	-3.66276	-0.93058
H	-4.96743	-1.52863	-0.78269
C	-2.94408	-4.67525	0.14516
H	-1.59521	-3.33432	1.13342
C	-4.11074	-4.79984	-0.59750
H	-5.74938	-3.74967	-1.50531
H	-2.37531	-5.55835	0.41554
H	-4.45675	-5.77846	-0.91102
C	4.53210	-0.29824	-0.69471
H	4.10190	-0.65461	-1.63645
H	4.86747	0.71743	-0.92823
C	5.73120	-1.16155	-0.30650
H	6.20273	-0.77753	0.60302
H	5.39027	-2.17892	-0.08040
C	6.74992	-1.22427	-1.40483
H	6.39874	-1.62020	-2.35660
C	8.01112	-0.83315	-1.29831
H	8.70101	-0.90183	-2.13165
H	8.40678	-0.42830	-0.37133
H	0.69269	3.40517	0.71796

26			
**27**			
C	2.86974	-0.68913	-0.00001
C	4.12482	1.37845	0.00011
C	5.27683	0.63269	0.00009
C	5.25439	-0.76260	0.00001
C	4.01965	-1.45331	-0.00003
C	0.76816	0.12188	0.00001
C	1.61434	1.18051	0.00008
H	4.06492	2.45463	0.00017
H	6.22386	1.15819	0.00012
H	6.18197	-1.31853	-0.00001
H	3.95601	-2.53174	-0.00009
H	1.42121	2.23699	0.00013
N	2.90747	0.68197	0.00006
N	1.55560	-1.02413	-0.00004
C	-0.69836	0.04643	-0.00001
C	-1.46811	1.17148	0.00002
C	-1.37259	-1.25039	-0.00004
C	-2.87662	1.14360	-0.00000
C	-2.81417	-1.23241	-0.00006
C	-3.54528	-0.06727	-0.00004
O	-0.76165	-2.33506	-0.00006
F	-0.91447	2.38870	0.00005
F	-3.54831	2.28125	0.00002
F	-4.87350	-0.08987	-0.00006
F	-3.44867	-2.39034	-0.00010
H	1.15157	-1.95114	-0.00009

26			
**28**			
C	-4.08489	-1.22814	-0.07406
C	-4.54393	0.07341	-0.08319
C	-3.62964	1.12906	-0.03101
C	-2.22692	0.91692	0.06397
C	-1.75976	-0.47227	0.06581
C	-2.68963	-1.48309	-0.02252
H	-4.77524	-2.06194	-0.11466
H	-5.60642	0.28030	-0.13517
H	-3.96462	2.15958	-0.00650
H	-2.34925	-2.51371	-0.06134
C	-0.35917	-0.78016	0.13486
H	-0.03730	-1.78450	0.37561

C	1.94828	0.02152	-0.04757
C	2.72202	1.18973	-0.08285
C	2.58853	-1.22254	0.00877
C	4.10104	1.11231	-0.05774
H	2.22646	2.15430	-0.12181
C	3.97240	-1.28230	0.03295
H	2.01611	-2.14142	0.01312
C	4.73858	-0.12391	0.00227
H	4.68550	2.02515	-0.08281
H	4.45834	-2.25100	0.07161
H	5.82036	-0.18235	0.02181
O	-1.41064	1.87794	0.16405
H	0.16213	1.10433	-0.13460
N	0.56966	0.15492	-0.08512

25			
**29**			
C	1.94998	-0.74372	0.00004
C	1.93113	0.65191	-0.00027
C	3.11609	1.37504	-0.00023
C	4.29938	0.64072	0.00014
C	4.30232	-0.75204	0.00042
C	3.10901	-1.47974	0.00037
C	-0.15932	-0.11945	-0.00033
H	3.12085	2.45774	-0.00045
H	5.24486	1.17044	0.00019
H	5.24609	-1.28364	0.00068
H	3.09289	-2.56203	0.00058
C	-1.57268	-0.22592	-0.00015
C	-2.36285	1.01359	0.00018
C	-2.21811	-1.44566	-0.00028
C	-3.77173	0.88167	0.00030
C	-3.63871	-1.52274	-0.00007
H	-1.63782	-2.36107	-0.00054
C	-4.40244	-0.37352	0.00019
H	-4.34719	1.79996	0.00059
H	-4.10939	-2.49865	-0.00013
H	-5.48413	-0.43518	0.00033
N	0.60244	0.99987	-0.00064
O	0.65627	-1.20669	-0.00017
O	-1.77525	2.13330	0.00040
H	0.11621	1.89969	-0.00058

25			

**30**			
C	-2.11428	0.65058	0.13706
C	-1.87790	-0.61140	-0.41622
C	-2.93622	-1.48387	-0.64224
C	-4.22001	-1.07793	-0.30724
C	-4.45410	0.18160	0.23573
C	-3.39755	1.05594	0.46166
C	0.28559	0.24154	-0.39071
H	-2.75843	-2.46124	-1.07672
H	-5.05110	-1.75248	-0.47773
H	-5.46349	0.48412	0.48747
H	-3.57421	2.03447	0.89283
C	1.74636	0.09966	-0.30331
C	2.32577	-0.50667	0.88504
C	2.57674	0.50457	-1.33181
C	3.76987	-0.60644	0.95976
C	3.95723	0.37921	-1.22728
H	2.13934	0.94015	-2.22227
C	4.54835	-0.17468	-0.06969
H	4.18941	-1.03813	1.86078
H	4.58995	0.71727	-2.03917
H	5.62831	-0.25350	-0.01172
N	-0.54085	-0.83826	-0.69804
O	1.59291	-0.95057	1.78629
H	-0.17303	-1.77549	-0.62930
S	-0.60895	1.54154	0.36670

24			
**31**			
C	-0.76847	-0.15669	0.00004
C	0.66289	-0.10719	-0.00015
C	1.48978	-1.33036	-0.00052
C	-1.54104	1.01423	-0.00029
C	1.28329	1.15771	-0.00005
C	0.48822	2.33791	-0.00040
C	-0.87704	2.25754	-0.00067
C	2.70386	1.23220	0.00048
C	3.48359	0.08864	0.00093
C	2.90671	-1.16711	0.00060
H	3.50093	-2.07246	0.00062
H	4.56426	0.18246	0.00148
H	-0.76141	-2.17160	0.00050
C	-2.74781	-1.50561	0.00083
C	-3.54821	-0.36889	0.00028

C	-2.97711	0.88009	-0.00013
H	-3.12898	-2.51582	0.00134
H	-4.62456	-0.48911	0.00015
H	3.17074	2.21059	0.00072
H	0.98298	3.30194	-0.00042
H	-1.47456	3.16325	-0.00103
H	-3.59111	1.77239	-0.00030
N	-1.39748	-1.36559	0.00116
O	0.97401	-2.47715	-0.00211

29<br>**32**			
C	4.42252	-1.46729	0.26821
C	3.14186	-2.01110	0.28448
C	2.03220	-1.21063	0.10026
C	2.17132	0.18250	-0.10394
C	3.48209	0.72021	-0.11672
C	4.57703	-0.09600	0.06533
C	1.05238	1.01459	-0.28723
C	-0.35094	0.55764	-0.33164
C	-0.93572	0.09153	-1.49861
C	-2.24797	-0.33696	-1.51503
H	-2.70544	-0.71837	-2.41905
C	-3.04212	-0.30142	-0.33341
C	-2.51907	0.17007	0.83305
C	-1.15062	0.63757	0.88154
H	5.28920	-2.10110	0.41268
H	3.00913	-3.07557	0.44384
H	3.60987	1.78387	-0.26655
H	5.57109	0.33781	0.05372
H	-3.08059	0.22964	1.75479
H	1.04678	-1.65744	0.11664
H	-0.34875	0.04231	-2.40808
O	1.27820	2.32595	-0.56330
O	-0.66652	1.12344	1.91670
H	0.49695	2.85455	-0.37710
O	-4.30102	-0.75881	-0.50292
C	-5.16322	-0.76633	0.61994
H	-6.10850	-1.17198	0.26965
H	-4.76494	-1.40049	1.41603
H	-5.31849	0.24582	1.00225

36<br>**33**			

C	-4.51097	-2.35417	0.00099
C	-3.12462	-2.22217	0.00018
C	-2.56245	-0.96067	-0.00034
C	-3.36821	0.19810	0.00000
C	-4.77029	0.03845	0.00086
C	-5.32804	-1.22428	0.00132
H	-4.95176	-3.34431	0.00135
H	-2.47306	-3.08774	-0.00010
C	-2.70478	1.43299	-0.00044
H	-5.39068	0.92583	0.00114
H	-6.40595	-1.33745	0.00196
C	-1.27427	1.51551	-0.00059
C	-0.51795	0.31012	-0.00073
O	-1.20167	-0.88718	-0.00146
O	-3.37696	2.58288	-0.00035
O	-0.77517	2.68257	-0.00046
H	-2.69351	3.28304	-0.00037
C	0.89307	0.16059	-0.00042
C	1.48065	-1.12815	-0.00079
C	1.78208	1.26404	0.00029
C	2.84294	-1.30452	-0.00060
H	0.83935	-1.99920	-0.00115
C	3.14237	1.08792	0.00047
H	1.37115	2.26327	0.00065
C	3.72437	-0.20162	-0.00009
H	3.23153	-2.31384	-0.00073
H	3.77107	1.96788	0.00101
N	5.08302	-0.37102	-0.00014
C	5.65010	-1.70251	-0.00053
H	5.34969	-2.27141	-0.88665
H	6.73506	-1.63222	-0.00186
H	5.35173	-2.27140	0.88635
C	5.96171	0.77974	0.00203
H	5.81186	1.40366	0.88912
H	6.99545	0.44280	0.00239
H	5.81323	1.40591	-0.88369

31			
**34**			
C	-4.36312	-0.94243	0.32245
C	-3.68590	0.25785	0.42211
C	-2.31854	0.32254	0.16576
C	-1.61932	-0.84622	-0.19405
C	-2.32185	-2.05951	-0.30161

C	-3.67502	-2.10178	-0.04375
C	-1.63031	1.62801	0.24805
C	-0.21004	-0.78795	-0.44137
C	0.51212	0.42702	-0.30316
C	-0.17961	1.63380	-0.03580
C	0.49680	2.84717	-0.05259
H	-0.06841	3.74690	0.15565
C	1.84589	2.89194	-0.33755
C	2.55760	1.71137	-0.56787
C	1.91951	0.49474	-0.50140
H	-5.42732	-0.98410	0.52368
H	-4.19620	1.17360	0.69414
H	-1.78658	-2.95612	-0.58429
H	-4.20737	-3.04266	-0.12616
H	2.37057	3.83966	-0.35681
H	3.62440	1.73504	-0.74445
O	-2.23246	2.65370	0.52225
O	0.35333	-1.92787	-0.85797
N	2.62152	-0.72337	-0.63790
C	3.28912	-1.18018	0.48498
H	1.28499	-1.77751	-1.12618
O	3.87955	-0.38968	1.19910
C	3.36592	-2.66890	0.64850
H	2.36540	-3.10514	0.69136
H	3.88088	-3.11094	-0.20635
H	3.90617	-2.90929	1.56160

19			
**35**			
C	2.48908	0.91619	0.01314
C	1.09083	0.97665	-0.01144
C	0.31262	-0.26067	-0.00228
C	0.99768	-1.48900	-0.00310
C	2.38042	-1.49852	0.01008
C	3.13381	-0.30511	0.02165
H	3.02778	1.85527	0.01179
H	0.41661	-2.39922	-0.00929
H	2.89860	-2.45031	0.01366
H	4.21557	-0.34978	0.03347
C	-3.02261	0.56076	0.03639
H	-3.91594	-1.48513	-0.04313
H	-3.83564	1.26834	0.07020
N	-1.75060	0.97460	0.06114
N	-1.03990	-0.21347	-0.00101

N	-1.82231	-1.34042	-0.03850
C	-3.05685	-0.83313	-0.01992
O	0.50425	2.14882	-0.05775
H	-0.50117	2.02229	-0.03329

21			
**36**			
C	-3.69320	0.26531	0.00001
C	-2.64361	1.17233	-0.00004
C	-1.33793	0.70992	-0.00003
C	-1.04240	-0.67485	0.00004
C	-2.12900	-1.56565	0.00009
C	-3.42971	-1.10353	0.00007
H	-0.39128	2.58928	-0.00035
H	-4.71454	0.62617	-0.00000
H	-2.83933	2.24016	-0.00008
H	-1.90390	-2.62490	0.00014
H	-4.25137	-1.81026	0.00011
C	1.22611	-0.23897	0.00008
C	1.01595	1.12985	-0.00009
N	0.23259	-1.15384	0.00009
N	-0.25076	1.59014	-0.00009
O	1.95499	2.05374	-0.00009
C	2.60690	-0.72909	0.00004
O	2.80111	-1.99362	-0.00046
N	3.62465	0.06473	0.00041
H	4.52947	-0.39710	0.00044
H	2.83811	1.59657	0.00024

19			
**37**			
C	0.10337	3.56360	3.66249
C	-1.26405	3.60118	3.75290
C	-1.98784	2.47427	4.26557
C	-1.27509	1.36469	4.66016
C	0.14296	1.32891	4.56514
C	0.88955	2.42866	4.06174
C	0.60142	4.76927	3.13504
C	-0.50850	5.71563	2.83784
C	-1.77887	4.92568	3.25557
H	-3.06871	2.50155	4.33807
H	-1.79638	0.49916	5.05124
H	0.68861	0.44689	4.88048
H	-0.52328	5.99747	1.77847

H	-0.39827	6.65225	3.39687
O	1.87854	5.03173	2.92937
O	2.14708	2.48383	3.94231
H	2.37590	4.22388	3.21781
H	-2.34567	5.44260	4.03562
H	-2.46963	4.78701	2.41856

23			
**38**			
C	-2.32500	-0.67032	-0.01693
C	-0.92886	-0.71495	-0.01669
C	-0.22402	-1.94953	-0.01693
C	-1.09312	-3.09813	-0.01735
C	-2.54091	-3.04457	-0.01759
C	-3.15581	-1.79503	-0.01740
H	-2.79439	0.30837	-0.01678
H	-0.35452	0.20402	-0.01631
H	-4.23267	-1.68240	-0.01759
C	-0.65150	-4.44923	-0.01766
C	-1.78878	-5.26388	-0.01804
C	-1.94598	-6.66514	-0.01847
C	-3.21170	-7.17719	-0.01888
C	-4.36504	-6.32745	-0.01889
C	-4.25530	-4.96699	-0.01847
C	-2.96876	-4.38393	-0.01806
H	-1.07516	-7.30925	-0.01850
H	-3.36022	-8.25078	-0.01923
H	-5.34626	-6.78819	-0.01924
H	-5.13862	-4.33924	-0.01845
O	0.62171	-4.80145	-0.01756
O	1.02031	-2.13323	-0.01663
H	1.13908	-3.95240	-0.01731

29			
**39**			
C	-2.48668	-0.76701	0.14017
C	-1.07642	-0.76935	0.16248
C	-0.30665	-1.97874	0.08696
C	-1.09990	-3.15143	-0.01062
C	-2.50468	-3.15582	-0.03341
C	-3.22351	-1.93189	0.04387
H	-3.00287	0.18453	0.20098
H	-0.53670	0.16836	0.23907
H	-4.30631	-1.91062	0.02817

C	-0.61957	-4.47010	-0.09878
C	-1.73880	-5.35973	-0.18123
C	-1.85150	-6.71866	-0.28174
C	-3.14155	-7.31713	-0.34584
C	-4.31548	-6.50031	-0.30533
C	-4.17977	-5.09258	-0.20095
C	-2.93103	-4.52925	-0.14005
H	-0.96930	-7.34880	-0.31341
H	-5.07283	-4.47604	-0.17040
O	0.66140	-4.79282	-0.10179
O	0.95302	-2.04595	0.10218
H	1.16014	-3.93949	-0.03252
C	-4.55363	-9.28001	-0.51097
C	-5.70734	-8.47683	-0.47110
C	-5.58483	-7.11365	-0.37015
C	-3.30278	-8.71246	-0.44976
H	-4.65261	-10.35676	-0.59074
H	-6.68790	-8.93545	-0.52009
H	-6.47022	-6.48676	-0.33879
H	-2.41888	-9.34063	-0.48124

19			
**40**			
C	-2.43488	-0.61421	0.14956
C	-1.03303	-0.79033	0.15080
C	-0.44765	-2.06750	0.07223
C	-1.32977	-3.23411	-0.01285
C	-2.69483	-3.03398	-0.01242
C	-3.25037	-1.71942	0.06934
H	-2.85670	0.38117	0.21132
H	-0.35937	0.05611	0.21300
H	-4.32850	-1.61161	0.06666
C	-0.73630	-4.52677	-0.09235
O	0.55744	-4.73365	-0.09602
O	0.81564	-2.24824	0.07205
H	0.97374	-3.79940	-0.03015
H	-3.36114	-3.88566	-0.07494
O	-1.52327	-5.58929	-0.16788
C	-0.90454	-6.87564	-0.24667
H	-1.72529	-7.58514	-0.29789
H	-0.28258	-6.94634	-1.13852
H	-0.29434	-7.06084	0.63679

25			

**41**			
C	0.36993	-2.78728	-0.05890
C	-1.04278	-2.77167	-0.16404
C	-1.77927	-1.65998	0.16775
C	-1.07061	-0.55014	0.65443
C	0.32316	-0.54072	0.74961
C	1.06040	-1.65684	0.39384
C	0.75223	-4.07694	-0.48470
C	-0.46947	-4.75509	-0.80512
H	-1.62776	0.33043	0.95094
H	0.82499	0.34948	1.10962
H	2.14229	-1.65461	0.46961
H	1.75103	-4.48393	-0.54978
H	-3.47920	-3.33384	1.14799
C	-3.26118	-1.63072	0.01389
C	-3.93228	-0.76744	-0.78693
C	-5.34523	-0.77081	-0.85777
H	-3.35860	-0.06476	-1.37799
C	-5.35752	-2.58522	0.72391
C	-6.03359	-1.71679	-0.07184
H	-5.87306	-0.08292	-1.50091
H	-5.83702	-3.33214	1.33906
H	-7.11532	-1.77034	-0.09259
N	-1.53186	-4.01659	-0.62337
N	-3.97132	-2.52306	0.81266
H	-0.55153	-5.77550	-1.15862

17<br>**42**			
C	-1.95447	-0.21702	0.05994
C	0.07993	1.02531	-0.16787
C	-1.98121	2.18319	0.04936
C	-2.66392	0.99188	0.13029
H	-3.74220	0.97999	0.24747
C	-2.52825	-1.58887	0.12971
H	-3.61090	-1.53885	0.24718
H	-2.31452	-2.16769	-0.77591
H	-2.12417	-2.15827	0.97431
C	-2.60660	3.53592	0.10952
H	-2.39915	4.10768	-0.80074
H	-3.68751	3.45986	0.22732
H	-2.20931	4.11772	0.94749
N	-0.60037	2.15538	-0.10044
N	-0.59331	-0.08050	-0.08864

S	1.82490	0.98863	-0.35732
H	1.93107	2.33032	-0.37620

28			
**43**			
C	-4.87036	-3.39266	0.20018
C	-3.50603	-3.32622	0.10648
C	-2.83667	-2.09291	0.02886
C	-3.56587	-0.87024	0.07760
C	-4.99448	-0.97486	0.08492
C	-5.60895	-2.20208	0.16225
C	-1.41891	-2.05760	-0.12574
C	-2.82592	0.34635	0.10704
C	-1.43630	0.36561	-0.11650
C	-0.75535	-0.88777	-0.23778
C	-0.74671	1.58596	-0.19328
H	0.31944	1.58544	-0.37644
C	-1.44477	2.79447	-0.02327
C	-2.76748	2.77442	0.27503
H	-0.88350	-2.99966	-0.17314
H	-5.38080	-4.34512	0.26999
H	-2.91415	-4.23538	0.08626
H	-6.69208	-2.24998	0.16185
H	0.31862	-0.87283	-0.38677
H	-0.93716	3.74666	-0.10664
H	-3.35708	3.66036	0.45937
N	-3.44555	1.57366	0.36447
C	-5.89512	0.19326	-0.13847
H	-4.29180	1.58292	0.90155
C	-6.67304	0.71631	0.89949
N	-7.27210	1.15231	1.78732
C	-6.06076	0.72536	-1.42095
N	-6.17241	1.15875	-2.48646
